# Coupled Above‐ and Belowground Ecosystem Stability Worldwide

**DOI:** 10.1002/advs.202517411

**Published:** 2026-03-09

**Authors:** Zexin Meng, Huiwen Li, Yiping Wu, Peter B. Reich, Nico Eisenhauer, Diego Abalos, Daniel Revillini, Shuguang Liu, Wende Yan, Ji Chen, Alexey Voinov, Zhifeng Yang, Ying‐Ping Wang, Fubo Zhao, Linjing Qiu, Jingfeng Xiao, Shantao An, Guopeng Liang, Manuel Delgado‐Baquerizo

**Affiliations:** ^1^ Institute of Global Environmental Change; Department of Earth & Environmental Science School of Human Settlements and Civil Engineering Xi'an Jiaotong University Xi'an China; ^2^ School of Soil and Water Conservation Central South University of Forestry and Technology Changsha China; ^3^ Shaanxi Key Laboratory of Qinling Ecological Intelligent Monitoring and Protection, School of Life Science and Technology Northwestern Polytechnical University Xi'an China; ^4^ Institute For Global Change Biology, and School for the Environment and Sustainability University of Michigan Ann Arbor Michigan USA; ^5^ Department of Forest Resources University of Minnesota St. Paul Minnesota USA; ^6^ Hawkesbury Institute for the Environment Western Sydney University Penrith New South Wales Australia; ^7^ German Centre for Integrative Biodiversity Research (iDiv) Halle‐Jena‐Leipzig Leipzig Germany; ^8^ Institute of Biology Leipzig University Leipzig Germany; ^9^ Department of Agroecology Aarhus University Tjele Denmark; ^10^ Laboratorio De Biodiversidad y Funcionamiento Ecosistémico Consejo Superior De Investigaciones Científicas (CSIC) Instituto de Recursos Naturales y Agrobiología de Sevilla (IRNAS) Sevilla Spain; ^11^ School of Ecology Hainan University Haikou China; ^12^ Department of Civil Engineering The University of Hong Kong Hong Kong China; ^13^ Faculty of Engineering Technology University of Twente Enschede Netherlands; ^14^ State Key Laboratory of Regional Environment and Sustainability School of Environment Beijing Normal University Beijing China; ^15^ CSIRO Environment Clayton South Victoria Australia; ^16^ Earth Systems Research Center Institute For the Study of Earth, Oceans, and Space University of New Hampshire Durham New Hampshire USA; ^17^ Department of Ecology & Evolutionary Biology Yale University New Haven Connecticut USA

**Keywords:** above‐ and belowground coupling, arid environments, climatic fluctuations, ecosystem stability, soil respiration

## Abstract

Upholding stable terrestrial ecosystems is integral to supporting climate regulation and planetary security. Yet, while aboveground ecosystem stability is widely described, global‐scale patterns in belowground ecosystem stability and how it connects to aboveground stability remain virtually unknown. Here, we assembled a global dataset including high‐resolution information on annual estimates of soil respiration from 4,544 communities and associated aboveground ecosystem productivity over the past four decades (1985–2018). We found that ecosystems with greater stability in aboveground productivity had greater long‐term stability in soil respiration, with a positive and significant connection between above‐ and belowground stability being especially strong in arid environments. Stable temperatures played a crucial role in reinforcing the stability and coupling of above‐ and belowground ecosystems. Our work provides new evidence of, and insights into, the local to global connections of stability of above‐ and belowground biological activity, and identifies a fundamental role of temperature stability in maintaining this stability under a changing climate.

## Graphical abstract

Are the worlds above and below our feet in sync? This global exploration reveals an entangled fate between above‐ and belowground ecosystem stability. It identifies arid regions as hotspots for this coupling and highlights temperature stability as a vital safeguard for maintaining ecosystem balance across our warming planet.

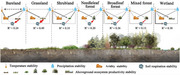

## 1 Introduction

Stable ecosystems ensure a consistent and reliable flow of resources that support both above‐ and belowground communities [1]. This stability, typically defined as the ratio of the mean to the standard deviation of an ecosystem process over time, reflects the reliability of biological activities [1]. By maintaining such stability in ecosystem‐level productivity, terrestrial biomes can continuously provide critical services, including carbon sequestration, nutrient cycling, and climate regulation. To date, aboveground ecosystem stability has been intensively documented over the last few decades, using plant productivity, biomass, or leaf area index as indicators [2, 3]. However, much less is known about the global patterns and drivers of belowground ecosystem stability, and whether they are coupled with aboveground ecosystem stability.

Soil carbon dynamics serve as a critical indicator of belowground ecosystem stability. As the largest terrestrial organic carbon pool, soils support key ecosystem services such as climate regulation, soil fertility, and food security [4, 5]. Its stability is primarily reflected in the constancy of soil respiration that encompasses carbon losses from multiple belowground components, including root respiration, microbial decomposition, and soil fauna activity [6]. At the global scale, soil respiration releases 72.6–110 Pg C per year, several times that of anthropogenic CO_2_ emissions (∼11 Pg C yr^−1^) [7, 8, 9]. Even small changes in soil respiration could result in substantial impacts on atmospheric CO_2_ concentrations and modulate the trajectory of global climate change [10]. Thus, maintaining the stability of this massive carbon flux is critical for climate mitigation and planetary security. Recent modelling efforts have yielded global and multi‐decadal estimates of soil respiration, providing deeper insights into its magnitude and spatial patterns [9, 10, 11, 12, 13, 14, 15, 16]. However, these studies have rarely addressed the temporal stability of soil respiration per se. Consequently, a comprehensive understanding of the global belowground stability patterns across diverse biomes and the environmental drivers governing their sensitivity to climatic fluctuations remains elusive.

Beyond understanding belowground stability in isolation, a second major gap concerns its coupling with aboveground stability. This inter‐system link is fundamental to the functional integrity of terrestrial ecosystems, as above‐ and belowground components are integrated through feedback loops that govern key ecosystem processes [17]. Specifically, plant productivity reflects the capacity of plant communities to fix atmospheric carbon, while soil respiration represents the return flux of carbon from soils to the atmosphere [6, 10]. Although experimental and observational studies have shown strong correlations between the magnitude of these fluxes [18, 19, 20, 21, 22], it remains unclear whether, and where, belowground ecosystem stability covaries with that of aboveground ecosystems, and how this link is modulated by environmental gradients. This uncertainty is particularly acute in vulnerable regions such as drylands, which cover nearly half of the Earth's land surface and support one‐third of the global population [22, 23, 24]. Accordingly, up to now, the interdependence of above‐ and belowground stability has been largely overlooked, primarily because prior research has focused on these components in isolation. Understanding the coupled stability of these two subsystems is therefore essential for forecasting the resilience of climate regulation and ecosystem security in a warming world.

**FIGURE 1:**
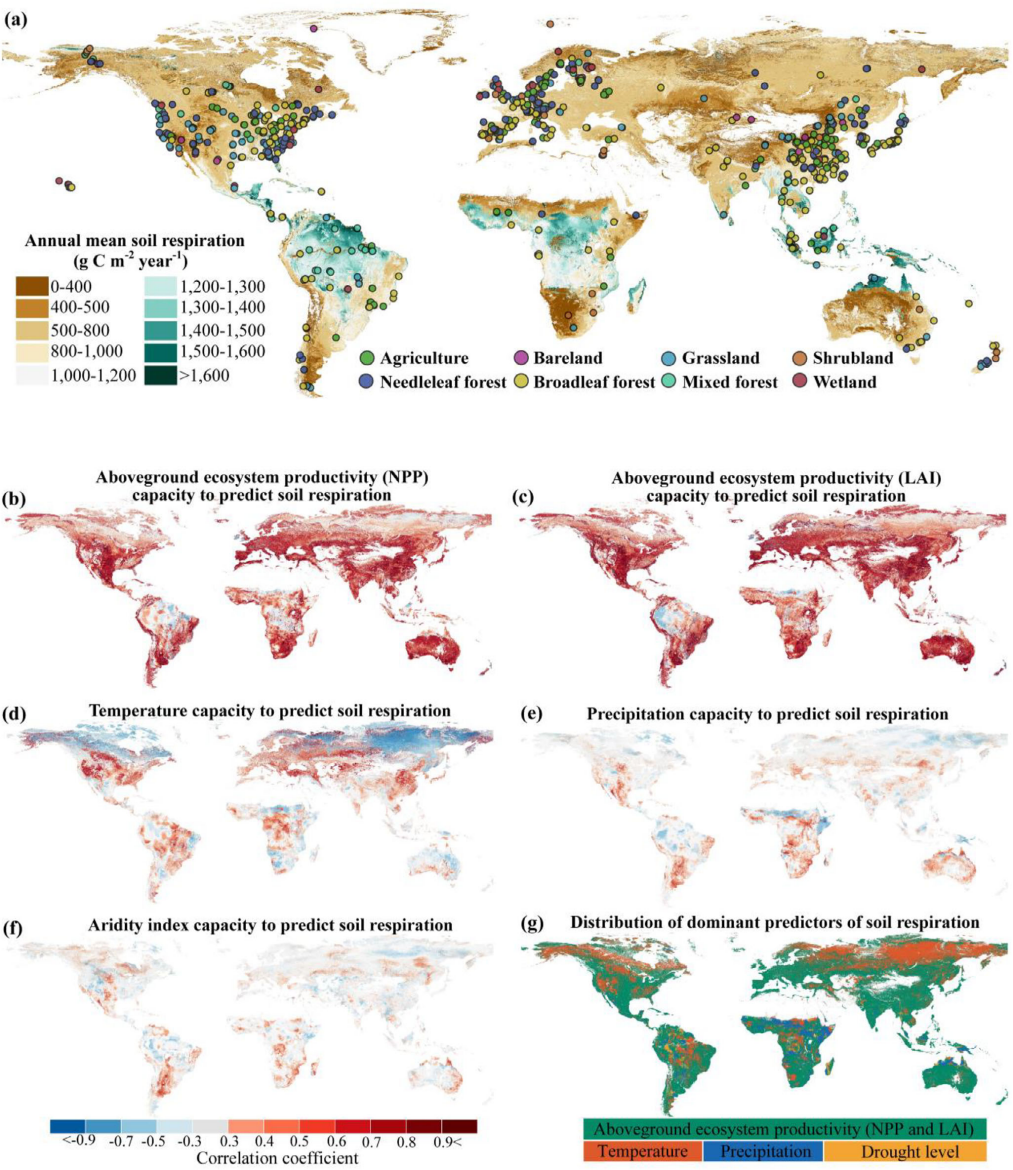
Global soil respiration and its predictors over the last four decades. (a) Global pattern of mean annual soil respiration over the last four decades at a 1 km^2^ spatial resolution, along with the locations of n = 4,544 communities (circles). A version of the map without observation points is provided in (Figure S7a). (b–f) Pearson correlation coefficient (R) between annual soil respiration and its predictors: aboveground ecosystem productivity NPP (b), aboveground ecosystem productivity LAI (c), temperature (d), precipitation (e), and aridity index (f). R = ±0.3 corresponds to the *p* < 0.05 significance level. (g) Spatial distribution of the dominant predictors of temporal variation in annual soil respiration, defined as the predictors that contribute the most to the increase (or decrease) in time in each soil respiration grid cell.

Here we synthesize a global database with high‐resolution, long‐term data on aboveground ecosystem productivity and soil respiration to quantify the relationships between above‐ and belowground ecosystem stability. Aboveground ecosystem dynamics are characterized using satellite‐based net primary productivity (NPP) to represent carbon fixation capacity and leaf area index (LAI) to reflect canopy structural integrity, spanning the period 1985 to 2018. We also use additional vegetation metrics such as the normalized difference vegetation index (NDVI), enhanced vegetation index (EVI), solar‐induced chlorophyll fluorescence (SIF), and biomass turnover rate to cross‐validate our findings and provide a comprehensive representation of aboveground ecosystem stability. To assess belowground ecosystem stability, we integrated 4,544 site‐year observations (Figure 1a) into a nonlinear model to generate a global 1 km^2^ resolution soil respiration product (1985–2018). This study aims to address three questions: (1) How can global spatiotemporal patterns of soil respiration be reliably reconstructed over multi‐decadal scales, and do their stability characteristics exhibit a robust coupling with aboveground vegetation metrics over multi‐decadal time scales? (2) How does this coupling vary along aridity gradients and across diverse biomes? and (3) to what extent do climatic conditions and their own stability mediate the strength of above‐ and belowground coupling? Closing these gaps will improve our capacity to understand and predict the stability of our terrestrial ecosystems under climate change.

## 2 Results and Discussion

**FIGURE 2:**
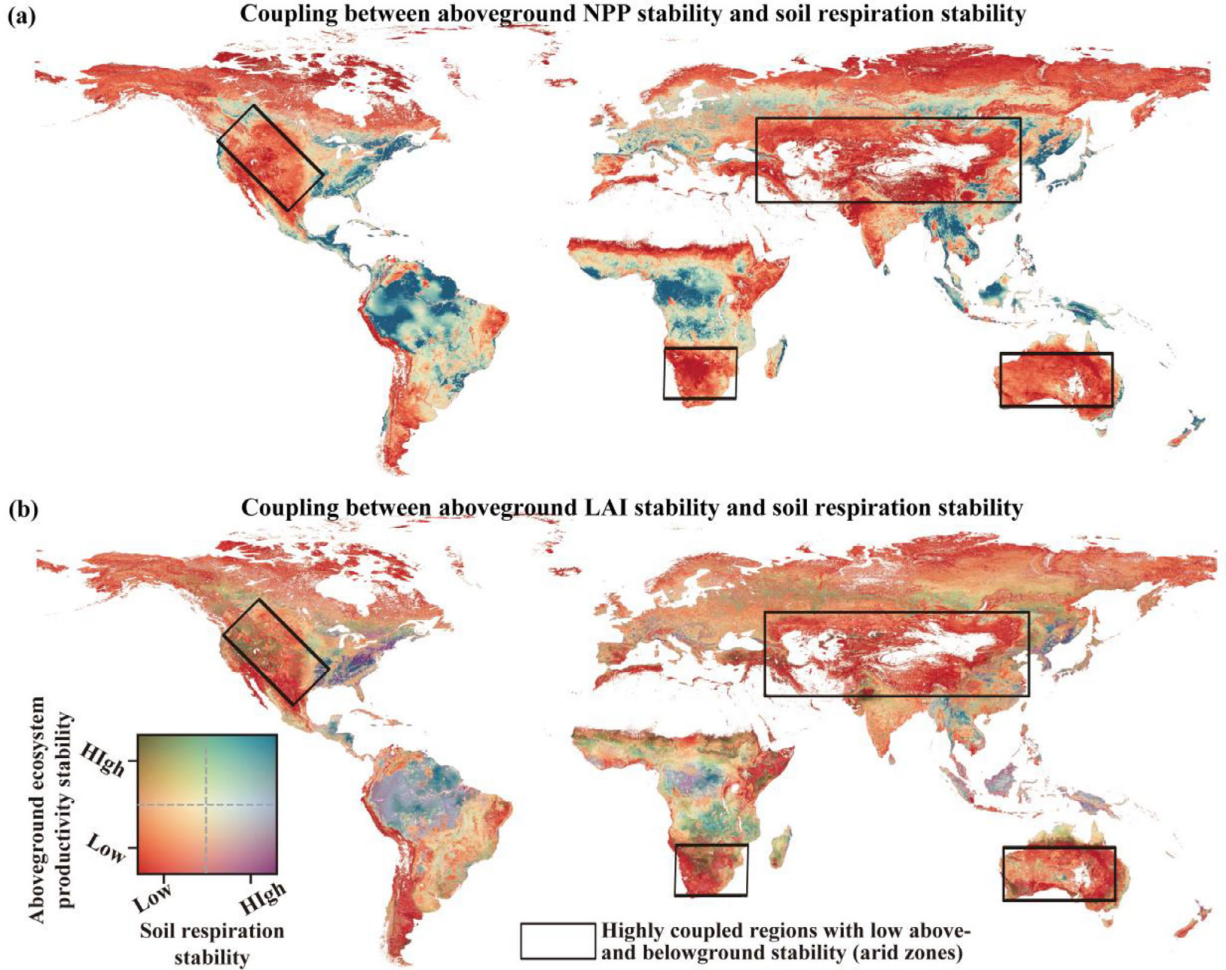
Coupling between above‐ and belowground ecosystem stability over the past four decades. (a, b) Global distribution of the overlap between aboveground (i.e., NPP and LAI) and belowground (soil respiration) interannual stability over the past four decades (1985–2018) at a 1 km^2^ resolution. Black boxes highlight arid regions with strong coupling and low stability in both aboveground and belowground ecosystems. Low and high values represent the 10th and 90th percentiles of the minimum and maximum values, respectively.

**FIGURE 3:**
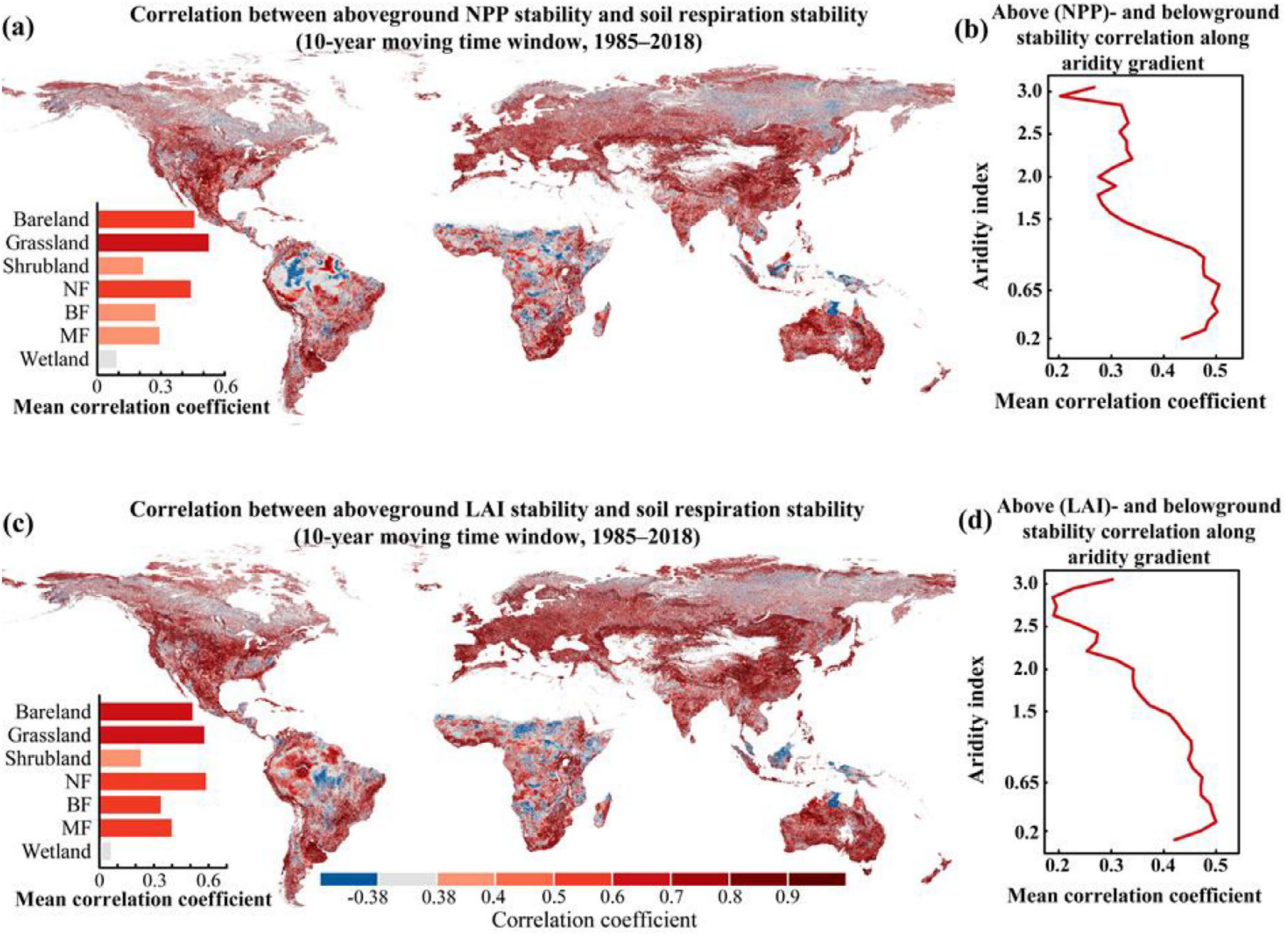
Coupling of above‐ and belowground stability across a 1‐year moving window (1985–2018). (a, c) Spatial correlation maps showing the relationship between soil respiration stability and aboveground ecosystem productivity NPP stability (a) and LAI stability (c), with stability calculated with a 10‐year moving window (e.g., 1985–1994, 1986–1995, and so forth until 2018). R = ±0.38 corresponds to the 0.05 significance level. The bar chart with each panel shows the mean correlation coefficient across different ecosystem types. NF: needleleaf forest; BF: broadleaf forest; MF: mixed forest. (b, d) Mean correlation coefficients of soil respiration stability with aboveground ecosystem productivity, NPP stability (b), and LAI stability (d) across the aridity gradient.

**FIGURE 4:**
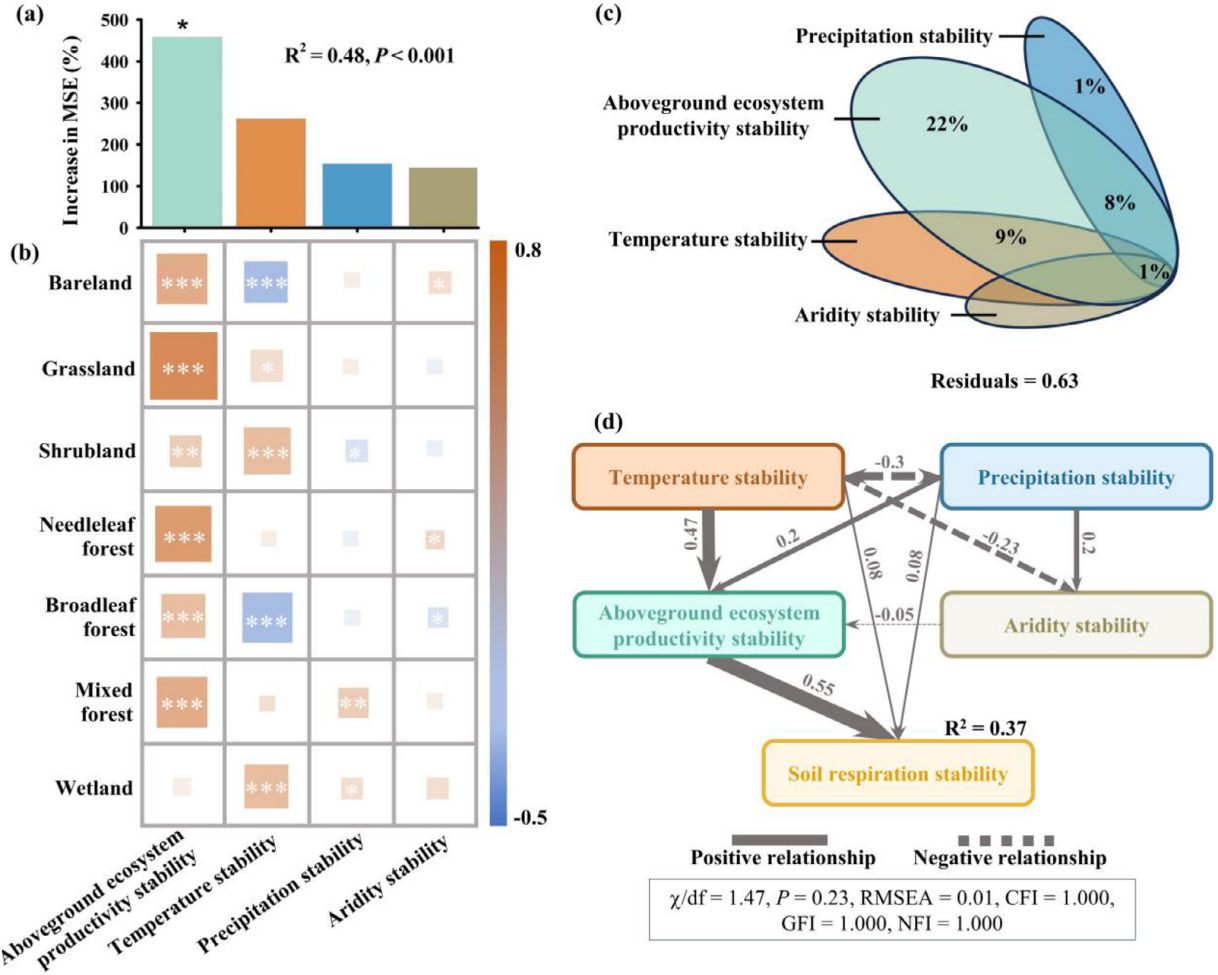
Coupling of aboveground and belowground stability and the role of climate stability in the coupling. All statistical analyses in this figure were conducted across global terrestrial grid cells. (a) Contribution of aboveground ecosystem productivity stability relative to climate stability as regulators of soil respiration interannual stability, based on a random forest model. Significance levels of each factor are as follows: ^*^ *p* < 0.05. (b) Partial correlations between soil respiration stability and aboveground ecosystem productivity stability, as well as climate stability across different ecosystem types. The *p*‐values were adjusted by false discovery rate: ^***^ *p* < 0.001, ^**^ *p* < 0.01, ^*^ *p* < 0.05. (c) Variance partition analysis quantifying the independent contributions of aboveground ecosystem productivity stability and climate stability to soil respiration interannual stability on a global scale. Only percent variation explained > 0 is shown (and thus areas shown are not to scale). (d) Structural equation models depicting the coupling relationship between aboveground ecosystem productivity stability and soil respiration stability, as well as the relative role of climate in this coupling. The width of black arrows is proportional to the strength of standardized path coefficients. R^2^ denotes the fraction of variance explained. Only significant pathways are shown (*p* < 0.05). Model fitness was evaluated using Chi‐square tests (χ^2^/df), Root Mean Square Error of Approximation (RMSEA), and Comparative Fit Index (CFI), with details presented in the bottom box. In all analyses, aboveground ecosystem productivity stability was represented by the first principal component of aboveground ecosystem productivity NPP stability and LAI stability to prevent overfitting.

**FIGURE 5:**
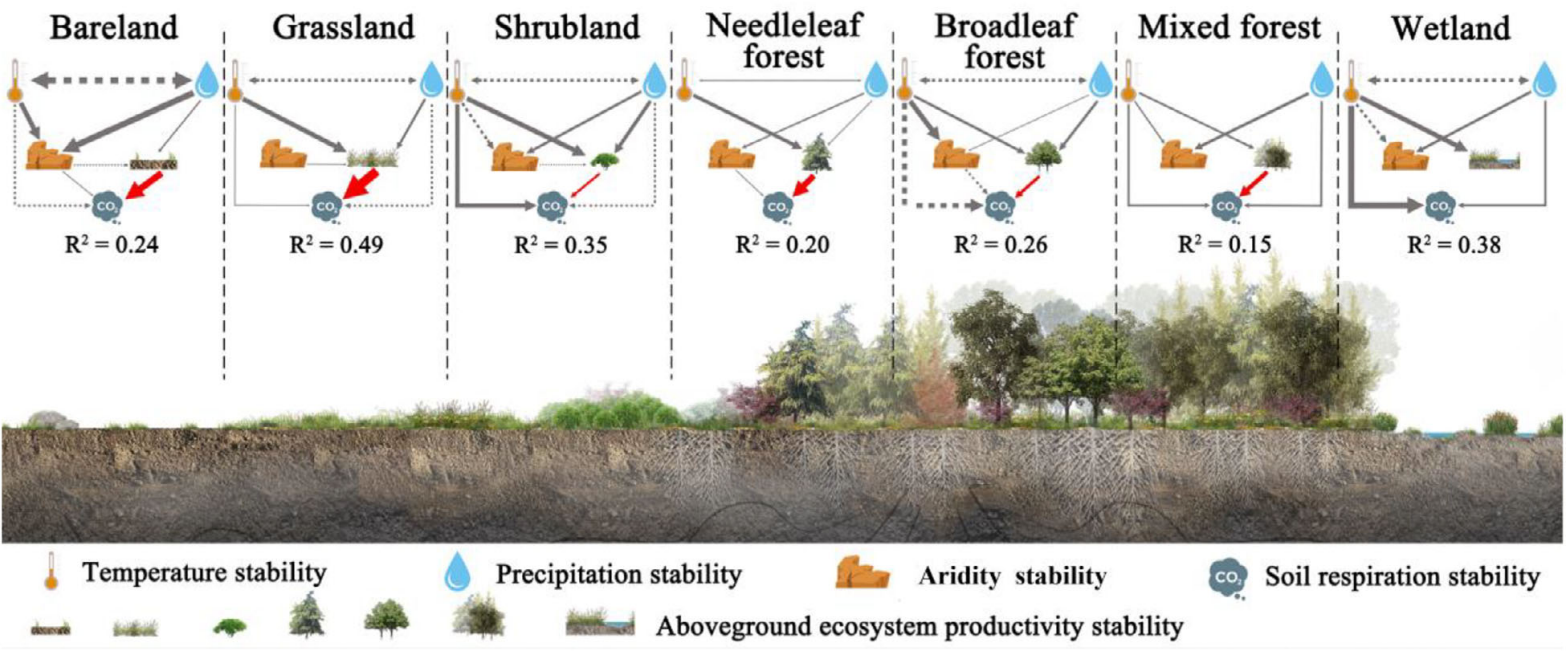
Coupling between aboveground and belowground stability at the ecosystem scale. All statistical analyses in this figure were conducted across global terrestrial grid cells. The width of the black arrows is proportional to the strength of the standardized path coefficients. R^2^ denotes the fraction of variance explained. Solid and dashed arrows represent positive and negative relationships, respectively. The red line represents the pathway between aboveground ecosystem productivity stability and soil respiration stability. To prevent overfitting in model analysis, aboveground ecosystem productivity stability was captured by the first principal component derived from NPP stability and LAI stability. Only significant pathways are shown (*p* < 0.05). The goodness‐of‐fit statistics for panels in each ecosystem type are reported in Table S9.

Our study demonstrates significant positive and long‐term coupling between above‐ and belowground ecosystem interannual stability across global environmental gradients (Figures 1, 2, 3). Greater stability in aboveground productivity supports more stable soil respiration, particularly in arid environments (Figures 2 and 3). Furthermore, thermal stability emerges as a primary determinant regulating the global coupling between above‐ and belowground ecosystem stability (Figures 4 and 5).

### 2.1 Coupled Above‐ and Belowground Ecosystem Stability Across the Globe

To investigate belowground ecosystem stability and its coupling with aboveground ecosystem stability, we generated a high‐resolution (1 km^2^) global soil respiration dataset spanning the period from 1985 to 2018 (Figure 1a). We first evaluated the predictive capacity of vegetation and climate variables for the interannual variation in soil respiration; our results revealed that biological drivers, such as NPP and LAI, exerted stronger predictive power than climatic factors, including temperature, precipitation, and the aridity index (Figure 1b–f). Vegetation metrics accounted for the largest share of soil respiration variance over the past four decades across most grid cells (Figure 1g). This predominance was also consistent across environmental gradients, with NPP and LAI retaining superior explanatory power across seven of the eight biomes and all aridity levels (Figures S1 and S2). These findings establish aboveground productivity as the primary driver of long‐term soil carbon efflux, reflecting a tight functional coupling driven by the rapid allocation of photosynthate to the rhizosphere [18]. Soil respiration and aboveground productivity also exhibit spatiotemporal coherence at the global scale, with high values converging in tropical regions and low values in arid ecosystems (Figure S3). Accordingly, given the tight metabolic coupling between both compartments, maintaining stable aboveground processes is paramount for sustaining belowground functional integrity and overall ecosystem resilience.

To further investigating the decadal relationship between above‐ and belowground ecosystem stability, we found that the interannual stability of soil respiration was positively and significantly associated with that of NPP and LAI globally (Figure 2). Extending the analysis to alternative vegetation metrics, including NDVI, EVI, SIF, and biomass turnover rate, also revealed that stability in canopy greenness, photosynthetic activity, and biomass renewal are all fundamental to maintaining stable belowground processes (Figure S4). More importantly, to ensure this coupling represents a robust ecological phenomenon rather than a modeling artifact, we reconstructed a global soil respiration dataset (1985–2018) using a Random Forest approach, excluding all vegetation‐related predictors. This independent reconstruction still yielded a strong coupling between the resulting soil respiration stability and the stability of all vegetation metrics (Figure S5). These results provide solid evidence and new insight that ecosystems with stable aboveground productivity maintain greater belowground stability across global environmental gradients.

Regions with higher above‐ and belowground stability are primarily concentrated in the tropics, notably the Amazon and Congo basins and parts of Southeast Asia (Figure 2; Figures S4 and S5). In these regions, thermal and hydrological constancy supports high species diversity and complex community structures, which collectively enhance the biophysical buffering capacity of rainforests against environmental fluctuations [25, 26]. However, this stability is vulnerable to severe disturbances such as large‐scale deforestation, prolonged drought, and fire [27, 28]. Such pressures disrupt ecosystem structure and functioning, accelerate soil degradation and trigger biodiversity loss, potentially pushing these systems toward irreversible regime shifts [27, 28]. This suggests that the observed coupling could be significantly reinforced during extreme climate events, such as severe droughts or heatwaves, which may synchronously suppress plant carbon fixation and microbial activity. This biological synchronization underscores the tight functional interdependence of both compartments, leading to simultaneous and severe reductions in above‐ and belowground processes. Given the increasing frequency and intensity of global change, our findings highlight the need for more attention to the coupled fate of above‐ and belowground stability, as their interconnected vulnerability becomes most prominent during these extreme events.

Ten‐year moving window analyses further demonstrated that temporal shifts in the stability of aboveground productivity and soil respiration were tightly coupled over a four‐decade study period. This temporal synchrony remained robust across the majority of biomes and aridity gradients (Figure 3), indicating that the interdependence of above‐ and belowground stability is not only a spatial pattern but also a persistent dynamic feature of terrestrial ecosystems. Ecosystems with higher productivity stability sustain greater long‐term soil respiration stability. Specifically, constant aboveground productivity ensures a steady rate of carbon fixation, which in turn stabilizes the accumulation and turnover of soil organic matter by providing a reliable influx of photosynthetic substrates [2, 29]. We cross‐validated these results using an independent Random Forest model and partial correlation analyses, which further confirmed that aboveground productivity stability remains the primary predictor of soil respiration stability, even when accounting for significant climatic influences (Figure 4a,b). Although temperature stability is a significant driver in five of seven biomes, six biomes retain robust positive associations between productivity and soil respiration stability, indicating a pervasive biological control (Figure 4b). This implies that the destabilization of vegetation can propagate belowground to disrupt soil communities and carbon cycling, heightening the vulnerability of terrestrial carbon dynamics to disturbance [30, 31, 32, 33]. For instance, shifts in resource availability have been shown to directly disrupt soil microbial communities, destabilizing soil carbon turnover [6, 34, 35, 36]. Such destabilization in turn affects nutrient availability, further reducing aboveground productivity and creating a feedback loop that undermines ecosystem resilience. Taken together, we argue that recognizing this coupled response is essential for accurately forecasting ecosystem shifts; the risks posed by climatic and anthropogenic disturbances are far greater than those estimated when above‐ and belowground components are evaluated in isolation.

Notably, the associations between above‐ and belowground stability are significantly weaker in wetland ecosystems and high‐latitude regions (Figure 3a,c). In wetlands, saturated conditions impose anaerobic constraints, making water table fluctuations instead of vegetation the primary determinant of soil metabolic rates [37]. Similarly, in high‐latitude regions such as northern Russia and Canada, soil respiration is primarily regulated by thermal kinetics and the mobilization of ancient organic matter during seasonal permafrost thaw [38]. Thus, our work provides solid evidence that the tight coupling of above‐ and belowground stability is a fundamental fate of terrestrial ecosystems, while also emphasizing that hydrological or thermal drivers exert a stronger influence on these dynamics in wetlands and high‐latitude regions.

### 2.2 Stronger Coupling of Above‐ and Belowground Stability in Arid Environments

We further found that the coupling between above‐ and belowground stability was strongest in arid environments (Figures 2 and 3; Figures S4 and S5). This was particularly evident in regions such as Central Asia, central Australia, the Midwestern United States, and southern Africa, which exhibited low stability in both aboveground productivity and soil respiration, yet showed the strongest coupling between the two domains (Figures 2 and 3; Figures S4 and S5). Drylands are vital for global climate and human well‐being, occupying over 40% of the Earth's land surface, and supporting approximately 38% of the global population, 90% of whom reside in developing countries [23, 24]. Temporal window analyses further confirmed that belowground stability became increasingly dependent on aboveground productivity under drier environments (Figure 3b,d). This intensified coupling stems from extreme water and nutrient scarcity, which compels the two subsystems into a state of obligatory interdependence: belowground heterotrophic life relies exclusively on ephemeral plant carbon inputs, while vegetation depends on microbial communities to safeguard soil structure and facilitate water retention [39, 40, 41, 42, 43]. Consequently, in these fragile ecosystems, even a small loss of vegetation, driven by land‐use change, overgrazing, or prolonged droughts, can trigger a much larger collapse in soil health than in other biomes [22, 44]. As climate stress intensifies, this tight dependency makes drylands especially vulnerable to future disturbances. Our work underscores that the systemic vulnerability of dryland ecosystems has been underestimated. Because previous studies often neglected stability and treated aboveground and belowground processes as decoupled entities, they failed to account for their intertwined fate and the true scale of potential functional collapse. There is an urgent need for strategies that prioritize the preservation of these interconnected processes to safeguard ecosystem resilience. Integrating the monitoring of both vegetation and soils, particularly within dryland regions, is therefore essential for refining climate impact assessments and for guiding evidence‐based conservation and restoration efforts.

### 2.3 Temperature Stability Shapes the Coupling Between Above‐ and Belowground Ecosystem Stability

Our global synthesis reveals that temperature stability acts as a primary modulator of above‐ and belowground stability and their coupling (Figures 4 and 5). Variance partitioning showed that aboveground productivity stability explained the largest unique share of soil respiration stability (22%), whereas interacting climate stability effects together accounted for about 18% of the explained variance (Figure 4c). This suggests that plant productivity stability serves as the primary direct link to soil respiration stability, whereas climatic influences operate largely through shared pathways by modulating plant‐soil interactions. Structural equation modeling (SEM) further supports this view and indicates that temperature stability affects soil respiration stability mainly by indirectly stabilizing aboveground productivity (Figure 4d). Biome‐level SEM further confirmed this (Figure 5), demonstrating that these relationships remain robust across diverse ecological zones. Stable temperatures promote steadier photosynthesis and reduce fluctuations in plant carbon inputs to soils [36, 45]. By limiting evaporative water loss, these mechanisms help maintain soil moisture and a steady substrate supply for microbes [34, 36, 46, 47], thereby strengthening the coupling between above‐ and belowground stability. Thus, these results underscore that temperature stability is a critical regulatory pillar of ecosystem reliability, suggesting that increasing climatic variability under a warming world may undermine terrestrial carbon cycle stability by disrupting the intrinsic coupling between above‐ and belowground processes.

Our study provides a comprehensive evaluation of the long‐term stability of soil respiration and plant productivity, demonstrating that their stabilities are tightly coupled across global environmental gradients. These findings advance our understanding of the intrinsic coupling between above‐ and belowground ecosystem stability, providing a mechanistic framework to better predict ecosystem resilience in an increasingly fluctuating climate. While this study represents a significant step forward, it also opens new avenues for future global change research aimed at refining the mechanistic understanding of ecosystem stability. First, future research should bridge the gap between soil respiration stability and net soil carbon sequestration, particularly under chronic, long‐term environmental change. Second, disentangling the contributions of microbial (heterotrophic) versus root (autotrophic) respiration could provide a more nuanced, comprehensive understanding of respiration stability. Third, while our multi‐proxy approach (using NPP, LAI, NDVI, EVI, SIF, and biomass turnover) confirms the robustness of aboveground ecosystem stability, we acknowledge that ‘community stability’ (species diversity) is another critical dimension that could not be explicitly quantified strictly due to the lack of continuous, high‐resolution global data. Thus, we advocate that future research should integrate species diversity metrics to unravel how community composition underpins this coupled stability. Finally, the stability measured here reflects temporal reliability, which is not equivalent to resilience against external disturbances. We emphasize that some regions identified as highly stable, such as degraded lands, may exist in a state that is both stable and degraded. In these ecosystems, soil respiration and productivity remain at consistently low levels, which does not represent high‐quality ecosystem services or security. We call for further research to integrate ecological resilience into the stability assessment framework to deepen our understanding of the fate of above‐ and belowground ecosystem coupling under global change.

In summary, our study provides new empirical evidence, and insights into, the strong positive coupling between the long‐term stability of aboveground and belowground ecosystems across global environmental gradients. This interdependency is particularly pronounced in dryland ecosystems, where belowground processes are strictly constrained by plant‐derived carbon inputs. Our findings identify temperature stability as a critical modulator of this link, a discovery that is essential for forecasting ecosystem responses to climate shifts. By highlighting the long‐term reliance of belowground stability on aboveground stability, we suggest that intensifying climate variability and anthropogenic pressures may destabilize these coupled ecosystem functions. These insights offer a framework to refine predictions of human‐induced environmental impacts and enhance the efficacy of global restoration and conservation strategies.

## 3 Materials and Methods

### 3.1 Aboveground Ecosystem

#### 3.1.1 Aboveground Ecosystem Productivity

We used satellite‐derived NPP and LAI as indicators of aboveground ecosystem productivity, because these metrics capture essential aspects of vegetation growth and structural capacity that underpin ecosystem stability. NPP directly reflects the rate of biomass accumulation and provides a robust measure of the ecosystem's ability to sequester carbon and sustain productivity over time. LAI quantifies the total leaf surface area per unit ground area, which is closely linked to photosynthetic capacity and energy balance, both critical for maintaining ecosystem function and resilience. These characteristics make NPP and LAI particularly suitable for assessing aboveground ecosystem stability, as they encompass both the carbon dynamics and structural attributes necessary for long‐term productivity and environmental interactions. We obtained 8‐day NPP and LAI products from the Global Land Surface Satellite (GLASS) project (http://www.glass.umd.edu/), aggregated them to annual values from 1985 to 2018, and used these time series to quantify temporal stability in aboveground ecosystem productivity. To test the robustness of our findings, we also quantified the stability of additional vegetation metrics, including NDVI, EVI, SIF, and a proxy for aboveground biomass turnover rate. NDVI and EVI were derived from MODIS MOD13A2 (https://ladsweb.modaps.eosdis.nasa.gov/missions‐and‐measurements/products/MOD13A2), SIF from the GOSIF dataset (https://globalecology.unh.edu/data/GOSIF.html), and the turnover rate proxy was calculated as annual NPP divided by aboveground biomass, the latter of which was derived from the dataset provided by Gao et al. [48].

### 3.2 Belowground Ecosystem

#### 3.2.1 Soil Respiration Database

The Global Soil Respiration Database (SRDB) compiles seasonal and annual soil respiration records from more than 10,000 published studies. We applied several filtering criteria to refine this dataset. Details on the different SRDB versions are documented in previous studies [49, 50]. Here, we used version 20200220a of SRDB, downloaded from https://github.com/bpbond/srdb. To ensure data consistency and accuracy, we used only the respiration records that (1) reported annual measurements, and (2) contained basic spatial and temporal information (longitude, latitude, and measurement years). Because the standard methods of soil respiration measurements, i.e., the use of infrared gas analyzers or gas chromatography, were not widely used before 1985, only a few soil respiration records were collected in 1961–1984. Thus, we only used soil respiration records after 1984 in this study. To minimize the influence of “extreme” values, we identified outliers as the measurements of soil respiration exceeding ± 3 standard deviations from the mean. A total of 4,544 annual soil respiration records (including summed autotrophic and heterotrophic respiration) from 1985–2018 were obtained, from 1,128 sites, which covered most ecosystem types, including bareland, cropland, grassland, shrubland, needleleaf forest, broadleaf forest, mixed forest, and wetland (Figure 1a).

#### 3.2.2 Environmental Predictors

To model global soil respiration, we used 28 environmental variables grouped into six categories: climate, vegetation, soil chemical properties, soil physical properties, soil microbial properties, and topography. Climate variables included mean annual temperature (MAT), mean annual maximum and minimum temperature, mean annual precipitation (MAP), incoming solar radiation, actual evapotranspiration (AET), and potential evapotranspiration (PET), obtained from the TerraClimate dataset (https://www.climatologylab.org/). For each climate variable, we also calculated a three‐year moving average to capture cumulative effects on soil respiration. We then performed principal component analysis (PCA) on the full set of climate variables and their three‐year averages and retained the first principal component (PC1) as a composite climate index. PC1 loadings and the proportion of variance explained are reported in Table S1.

Vegetation variables were characterized using global datasets from the GLASS project (http://www.glass.umd.edu/). We used annual NPP, LAI, and fractional vegetation cover (FVC). Carbon use efficiency (CUE) was calculated as the ratio of NPP to gross primary productivity (GPP), and water use efficiency (WUE) as NPP divided by AET. As for climate, we computed three‐year moving averages for all vegetation variables and then applied PCA to NPP, LAI, FVC, CUE, and WUE and their three‐year averages. The first principal component was retained as an integrated vegetation index, with PC1 loadings and explained variance shown in Table S2.

Soil chemical properties included soil organic carbon (SOC), total nitrogen (TN), and the C:N ratio in the 0–1 m layer, obtained from the SoilGrids250m project (https://maps.isric.org/). PCA on these three variables yielded a soil chemical index (PC1, Table S3). Soil physical properties were represented by sand, silt, and clay fractions, bulk density, volumetric soil water content, soil pH, and cation exchange capacity in the 0–1 m layer, also from SoilGrids250m. Their first principal component was used as a soil physical index (Table S4).

Soil microbial properties were represented by microbial biomass carbon, nitrogen, and phosphorus, upscaled to the global scale by Gao et al. [51]. PCA on these three variables yielded a microbial index (PC1, Table S5). Topographic variables included elevation using a digital elevation model (DEM), slope, and aspect derived from the ASTER Global DEM (https://asterweb.jpl.nasa.gov/GDEM.asp). PCA on elevation, slope, and aspect produced a topographic index (PC1, Table S6).

The original spatial resolution of the environmental datasets ranged from 30 m to 5 km. We resampled all variables to a common 1 km × 1 km grid using nearest neighbor resampling and aggregated daily, 8‐day, and monthly satellite products to annual values. Low‐quality observations were removed using the quality flags provided with each product. To stratify analyses by ecosystem type, we used the ESA global land cover product (https://viewer.esa‐worldcover.org/) and regrouped the original classes into eight ecosystem types: bareland, cropland, grassland, shrubland, needleleaf forest, broadleaf forest, mixed forest, and wetland.

In this study, we employed 28 ecosystem factors, including climate, vegetation, topography, soil physical, soil chemical, and soil microbial variables, to comprehensively and accurately model the global soil respiration dataset (Table S7).

#### 3.2.3 Non‐Linear Stepwise Regression Model

We used the geographic coordinates of the global soil respiration database to obtain corresponding climatic, vegetation, soil physical, soil chemical, soil microbial, and topographic indices for the sample points. We then applied a nonlinear stepwise regression model across different ecosystem types, yielding a high‐resolution, long‐term global soil respiration dataset. The formula for this method is shown in Equation (1):

(1) $$\begin{array}{ccc} Rs & = & \beta 0+\beta 1\;\times \;Cli+\beta 2\;\times \;Cli^{2}+\beta 3\;\times \;Plant+\beta 4\;\times Plant^{2}\\ & & +\beta 5\;\times \;Gra+\beta 6\;\times Gra^{2}+\beta 7\;\times \;Mic+\beta 8\;\times \;Mic^{2}\\ & & +\beta 9\;\times \;Phy+\beta 10\;\times Phy^{2}+\beta 11\;\times \;Chem\\ & & +\beta 12\;\times \;Chem^{2}+\varepsilon \end{array}$$

where soil respiration is annual soil respiration (Pg C year^−1^), β0 is the constant term of the regression equation, β1 to β12 are the regression coefficients, Cli represents the climate index, Plant is the vegetation index, Gra is the topographic index, Mic is the soil microbial index, Phy is the soil physical index, Chem is the soil chemical index, and ε is the error term.

#### 3.2.4 Model Validation

We evaluated model performance using a strict site‐independent cross‐validation strategy at both the global scale and across distinct ecosystem types. To prevent potential data leakage caused by spatial autocorrelation, we enforced a constraint that restricted all records from a unique Site ID to a single fold. This ensured that no site appeared simultaneously in both the training and validation sets. Within each ecosystem type, sites were partitioned based on this constraint, with approximately 80% of sites assigned to the training set and the remaining 20% held out for independent validation (Figure S6). Moreover, our approach was compared with previous global studies on soil respiration across multiple dimensions, including soil respiration rate estimates, temporal scale, spatial resolution, modeling factors, and modeling approaches. Notably, our model exhibits high operability and comprehensively integrates both biotic and abiotic modeling factors—such as climate, topography, vegetation, soil physicochemical properties, and microbial attributes—across a long‐term (1985–2018) and high spatial resolution (1 km^2^) scale (Table S8). It yields a global mean annual soil respiration of 86.34 Pg C yr^−1^, which lies within the previously reported range of 72.6–110 Pg C yr^−1^ (Table S8). The temporal and spatial patterns produced by our soil respiration product are also consistent with recent global estimates, such as those by Lu et al. [14] and Huang et al. [9], which further supports the robustness of our model (Figure S7). Additionally, Pearson correlation analysis was used to quantify the relationships between annual soil respiration, aboveground ecosystem productivity (NPP and LAI), and climatic factors, clarifying their relative predictive capacity to spatiotemporal dynamics of soil respiration.

### 3.3 Statistical Analyses

#### 3.3.1 Aboveground and Belowground Ecosystem Stability

We quantified stability of aboveground and belowground processes over 1985–2018 as the ratio of the mean to the standard deviation of annual values (µ/σ) for each 1 km^2^ grid cell [3, 46]. For aboveground ecosystems, stability was calculated for NPP and LAI, as well as for additional vegetation metrics (NDVI, EVI, SIF, and biomass turnover). Belowground stability was quantified analogously using annual soil respiration from our product.

In addition to calculating stability metrics over the entire four‐decade period (1985–2018), we applied a 10‐year moving window approach to further explore the long‐term coupling of aboveground and belowground ecosystem stability. For each successive decadal window (e.g., 1985–1994 to 2009–2018), stability metrics were derived from the mean‐to‐standard deviation ratio of annual ecosystem productivity and soil respiration. This time‐resolved perspective, integrated with full‐period assessments, enabled a multi‐scale evaluation of ecosystem stability dynamics, ensuring the robustness of our findings across both long‐term and decadal scales.

#### 3.3.2 Coupling between Above‐ and Belowground Ecosystem Stability

We mapped the global alignment between the stability of aboveground ecosystem productivity (NPP and LAI) and soil respiration to identify spatial relationships between aboveground and belowground ecosystem stability over the past four decades (1985–2018). To capture temporal dynamics, we calculated Pearson correlation coefficients between the stability metrics of aboveground (NPP and LAI) and belowground (soil respiration) processes across a 10‐year moving window, revealing how these stabilities evolved concurrently over time. These patterns were further analyzed across ecosystem types and aridity gradients to test the robustness of the observed coupling, with a particular focus on arid regions, which are especially vulnerable to global climate change and play a key role in planetary stability. Specifically, the aridity index was calculated as the ratio of annual MAP to annual PET for each year from 1985 to 2018.

Further verification of above‐ and belowground ecosystem stability coupling using a vegetation‐independent machine learning model: To cross‐verify the robustness of the strong coupling between above‐ and belowground ecosystem stability, we reconstructed the global soil respiration dataset (1985–2018) using a Random Forest algorithm. This model excluded all vegetation‐related variables, including NPP, LAI, and FVC, to test whether the coupling remains consistent when using a different modeling approach without vegetation parameters. We parameterized the model using abiotic variables categorized into the following groups: climate, soil physicochemical properties, microbial attributes, and topographic features. The Random Forest model was optimized with 5,000 decision trees. During the training phase, we implemented a ten‐fold cross‐validation to evaluate the model performance, which yielded a Pearson correlation coefficient (R) of 0.82, a Mean Absolute Error (MAE) of 215.36, and a Root Mean Square Error (RMSE) of 360.34 (Figure S8). This alternative soil respiration product was then used to re‐evaluate the coupling between above‐ and belowground stability.

#### 3.3.3 Coupling between Above‐ and Belowground Ecosystem Stability Across Different Environmental Contexts

To further validate the coupling between above and belowground ecosystem stability and elucidate the role of climate stability in mediating this relationship, we adopted a global grid‐based sampling approach. The global datasets were segmented into one‐degree resolution grids, with each grid serving as an independent sampling unit. This approach balanced data volume while ensuring a sufficient sample size to capture meaningful spatial patterns from the high‐resolution (1 km^2^) global dataset. Then, Random Forest (RF) was performed to explore the relative importance of aboveground ecosystem productivity and climate stability (i.e., temperature, precipitation, and aridity index) on soil respiration stability with the “*randomForest*” and “*importance*” function of the *randomForest* package [46]. Partial correlation analysis was used to further verify the role of aboveground ecosystem productivity stability relative to climate stability in determining soil respiration stability across different ecosystem types [36, 52]. The *varpart* function in the R package *vegan’* was used. The partial correlation coefficient represents the correlation of each pair of variables after statistically controlling for all the other variables. Additionally, Variance Partitioning Analysis was utilized to explore their explanatory proportions for the variations in the stability of soil respiration with the package *vegan* in R. Finally, the SEM analysis was conducted to further disentangle the coupling between aboveground ecosystem productivity and soil respiration stability, and to examine how climate stability mediates their interrelationship across global and different ecosystem types (Figure S9) [53]. We considered a good model fit when the chi‐square (χ^2^) test is low (≤2), the probability level (*P*) is high (>0.05), and the root mean square error of approximation is low (≤0.05). To prevent overfitting in site‐level data analysis, aboveground ecosystem productivity stability was captured by the first principal component derived from NPP stability and LAI stability. The geographic datasets were visualized using the mapping tools in ArcGIS 10.2 (ESRI 2013; Environmental Systems Research Institute, Redlands, CA, USA). The statistical analysis was performed in R version (v.4.0.0).

## Author Contributions

Conceptualization: Y.P.W., M.D.‐B., and Z.X.M.; Methodology: Y.P.W., Z.X.M., M.D.‐B., and H.W.L.; Investigation: Z.X.M., Y.P.W., M.D.‐B., and H.W.L.; Visualization: Z.X.M., H.W.L., M.D.‐B., and S.T.A.; Supervision: Y.P.W., M.D.‐B., P.B.R., and N.E.; Writing – original draft: Z.X.M., Y.P.W., M.D.‐B., P.B.R., and H.W.L.; Writing – review and editing: Y.P.W., Z.X.M., H.W.L., D.A., M.D.M.D.‐B.,‐B., S.G.L., A.V., Z.F.Y., Y.‐P.W., F.B.Z., L.J.Q., J.F.X., G.P.L., N.E., P.B.R., W.D.Y., and D.R. In particular, M.D.‐B. made fundamental contributions to the study design, data analysis, and the development of research findings. He also led the writing, extensive review and editing, and the visualization of the findings. At the same time, P.B.R. and N.E. also made substantial contributions to the experimental design, the conceptualization of research findings, and the manuscript writing and refinement.

## Conflicts of Interest

The authors declare no conflicts of interest.

## Supporting information




**Supporting File**: advs74741‐sup‐0001‐SuppMat.docx.

## Data Availability

We retrieved soil respiration data from version 20200220a of SRDB downloaded from (https://github.com/bpbond/srdb). The global land cover product was provided by the European Space Agency (https://viewer.esa‐worldcover.org/). The land use data was collected from the Resources and Environmental Science Data Center (https://www.ncdc.ac.cn). Climate variables, namely mean annual temperature, annual high temperature, annual low temperature, mean annual precipitation, solar radiation, and actual and potential evapotranspiration, were acquired from the TerraClimate dataset (https://www.climatologylab.org/). We collected the global leaf area index, net primary productivity, and fractional vegetation coverage products from available GLASS datasets (http://www.glass.umd.edu/). NDVI and EVI were derived from MODIS MOD13A2 (https://ladsweb.modaps.eosdis.nasa.gov/missions‐and‐measurements/products/MOD13A2), and SIF was obtained from the GOSIF dataset (https://globalecology.unh.edu/data/GOSIF.html). All soil variables were from the SoilGrids250m project (https://maps.isric.org/). The Digital Elevation Model (DEM) was downloaded from ASTER Global DEM (https://asterweb.jpl.nasa.gov/GDEM.asp). Source data are provided with this paper.
