# Supplementary material for: Coupled Above‐ and Belowground Ecosystem Stability Worldwide
Source: Adv Sci (Weinh). 2026 Mar 9;13(28):e17411. doi: 10.1002/advs.202517411 (PMC13185820; doi:10.1002/advs.202517411)
Supplement: Supplementary file 1 — Supporting File: advs74741‐sup‐0001‐SuppMat.docx. [file ADVS-13-e17411-s001.docx]

**Coupled above- and belowground ecosystem stability worldwide**

Zexin Meng^1,2#^, Huiwen Li^3#^, Yiping Wu^1,2*^, Peter B. Reich^4,5,6^, Nico Eisenhauer^7,8^, Diego Abalos^9^, Daniel Revillini^10^, Shuguang Liu^11^, Wende Yan^2^, Ji Chen^12^, Alexey Voinov^13^, Zhifeng Yang^14^, Ying-Ping Wang^15^, Fubo Zhao^2^, Linjing Qiu^2^, Jingfeng Xiao^16^, Shantao An^2^, Guopeng Liang^4,17^, and Manuel Delgado-Baquerizo^10^

^1^ Institute of Global Environmental Change, Department of Earth & Environmental Science, School of Human Settlements and Civil Engineering, Xi’an Jiaotong University, Xi’an 710049, China.

^2^ School of Soil and Water Conservation, Central South University of Forestry and Technology, Changsha 410004, China.

^3^ School of Ecology and Environment, Northwestern Polytechnical University, Xi’an 710129, China.

^4^ Institute for Global Change Biology, and School for the Environment and Sustainability, University of Michigan, Ann Arbor, Mi 48109, USA.

^5^ Department of Forest Resources, University of Minnesota, St. Paul, MN 55108, USA

^6^ Hawkesbury Institute for the Environment, Western Sydney University, Penrith, NSW 2753, Australia.

^7^ German Centre for Integrative Biodiversity Research (iDiv) Halle-Jena-Leipzig; Puschstrasse 4, 04103 Leipzig, Germany.

^8^ Institute of Biology, Leipzig University; Puschstrasse 4, 04103 Leipzig, Germany.

^9^ Department of Agroecology, Aarhus University, Tjele 8830, Denmark.

^10^ Laboratorio de Biodiversidad y Funcionamiento Ecosistémico. Instituto de Recursos Naturales y Agrobiología de Sevilla (IRNAS). Consejo Superior de Investigaciones Científicas (CSIC). Av. Reina Mercedes 10, E-41012 Sevilla, Spain.

^11^ School of Ecology, Hainan University, Haikou 570228, China.

^12^ Department of Civil Engineering, The University of Hong Kong, Hong Kong 999077, China.

^13^ Faculty of Engineering Technology, University of Twente, Enschede, 7500, AE, Netherlands.

^14^ State Key Laboratory of Regional Environment and Sustainability, School of Environment, Beijing Normal University, Beijing, 100875, China.

^15^ CSIRO Environment, Clayton South, Victoria 3168, Australia.

^16^ Earth Systems Research Center, Institute for the Study of Earth, Oceans, and Space, University of New Hampshire, Durham, NH, 03824, USA.

^17^ Department of Ecology & Evolutionary Biology, Yale University, New Haven, CT 06520-8106, USA.

^#^These authors contributed equally to this work.

Correspondence: Yiping Wu ([rocky.ypwu@gmail.com](mailto:rocky.ypwu@gmail.com), [yipingwu@xjtu.edu.cn](mailto:yipingwu@xjtu.edu.cn))

**This file includes:**

Tables S1 to S9

Figures. S1 to S9

Supplementary references

**Tables S1** Mean loadings of vegetation variables on the first principal component (PC1) and corresponding variance explained for the period 1985 to 2018.

| **Vegetation variables** | **Mean PC1 loading (min–max)** |
| --- | --- |
| NPP | 0.368 (0.362–0.374) |
| LAI | 0.370 (0.362–0.379) |
| FVC | 0.353 (0.346–0.359) |
| CUE | -0.268 (-0.282–-0.252) |
| WUE | 0.167 (0.073–0.240) |
| NPP_3y | 0.369 (0.363–0.375) |
| LAI_3y | 0.372 (0.363–0.380) |
| FVC_3y | 0.353 (0.346–0.359) |
| CUE_3y | -0.268 (-0.282–-0.252) |
| WUE_3y | 0.168 (0.095–0.230) |
| **Proportion of variance (PC1, %)** | **67.6 (65–70.5)** |

NPP, net primary productivity; LAI, leaf area index; FVC, fractional vegetation cover; CUE, carbon use efficiency; WUE, water use efficiency; NPP_3y, three-year mean NPP; LAI_3y, three-year mean LAI; FVC_3y, three-year mean FVC; CUE_3y, three-year mean CUE; WUE_3y, three-year mean WUE.

**Tables S2** Mean loadings of climate variables on the first principal component (PC1) and corresponding variance explained for the period 1985 to 2018.

| **Climate variables** | **Mean PC1 loading (min–max)** |
| --- | --- |
| MAT | 0.304 (0.301–0.308) |
| MAP | 0.177 (0.153–0.188) |
| Tmax | 0.303 (0.300–0.307) |
| Tmin | 0.302 (0.300–0.305) |
| Srad | 0.260 (0.256–0.263) |
| AET | 0.223 (0.208–0.232) |
| PET | 0.274 (0.272–0.276) |
| MAT_3y | 0.304 (0.301–0.308) |
| MAP_3y | 0.180 (0.168–0.186) |
| Tmax_3y | 0.303 (0.300–0.307) |
| Tmin_3y | 0.302 (0.299–0.306) |
| Srad_3y | 0.261 (0.259–0.265) |
| AET_3y | 0.225 (0.219–0.230) |
| PET_3y | 0.274 (0.272–0.279) |
| **Proportion of variance (PC1, %)** | **75.8 (74–77.1)** |

MAT, mean annual temperature; MAP, mean annual precipitation; Tmax, mean annual maximum temperature; Tmin, mean annual minimum temperature; Srad, solar radiation; AET, actual evapotranspiration; PET, potential evapotranspiration; MAT_3y, three-year mean MAT; MAP_3y, three-year mean MAP; Tmax_3y, three-year mean Tmax; Tmin_3y, three-year mean Tmin; Srad_3y, three-year mean SR; AET_3y, three-year mean AET; PET_3y, three-year mean PET.

**Tables S3** Loadings of soil chemical variables on the first principal component (PC1) and proportion of variance explained.

| **Soil chemical variables** | **Mean PC1 loading** |
| --- | --- |
| SOC | 0.66 |
| TN | 0.51 |
| C:N | 0.55 |
| **Proportion of variance (PC1, %)** | **0.74** |

SOC, soil organic carbon; TN, total nitrogen; C:N, soil carbon to nitrogen ratio.

**Tables S4** Loadings of soil physical variables on the first principal component (PC1) and proportion of variance explained.

| **Soil physical variables** | **Mean PC1 loading** |
| --- | --- |
| SM | 0.450 |
| BD | -0.489 |
| CEC | 0.392 |
| pH | -0.194 |
| Clay | -0.207 |
| Sand | -0.302 |
| Silt | 0.482 |
| **Proportion of variance (PC1, %)** | **0.43** |

BD, bulk density; SM, soil moisture; pH, soil pH; CEC, cation exchange capacity; Clay, clay content; Sand, sand content; Silt, silt content.

**Tables S5** Loadings of soil microbial variables on the first principal component (PC1) and proportion of variance explained.

| **Soil microbial variables** | **Mean PC1 loading** |
| --- | --- |
| MBC | 0.57 |
| MBN | 0.57 |
| MBP | 0.59 |
| **Proportion of variance (PC1, %)** | **0.93** |

MBC, microbial biomass carbon; MBN, microbial biomass nitrogen; MBP, microbial biomass phosphorus.

**Tables S6** Loadings of topographic variables on the first principal component (PC1) and proportion of variance explained.

| **Topographic variables** | **Mean PC1 loading** |
| --- | --- |
| Elevation | 0.71 |
| Slope | 0.71 |
| Aspect | 0.01 |
| **Proportion of variance (PC1, %)** | **0.50** |

**Tables S7** Description and data sources of the 28 environmental predictors used for modeling global soil respiration.

| Category | Variable Name | Abbreviation/Details | Data Source |
| --- | --- | --- | --- |
| Climate | Mean Annual Temperature | MAT | TerraClimate |
|  | Mean Annual Maximum Temperature | Tmax | TerraClimate |
|  | Mean Annual Minimum Temperature | Tmin | TerraClimate |
|  | Mean Annual Precipitation | MAP | TerraClimate |
|  | Solar Radiation | Srad | TerraClimate |
|  | Actual Evapotranspiration | AET | TerraClimate |
|  | Potential Evapotranspiration | PET | TerraClimate |
| Vegetation | Annual Net Primary Productivity | NPP | GLASS Project |
|  | Leaf Area Index | LAI | GLASS Project |
|  | Fractional Vegetation Cover | FVC | GLASS Project |
|  | Carbon Use Efficiency | Ratio of NPP to GPP | Derived from GLASS |
|  | Water Use Efficiency | Ratio of NPP to AET | Derived from GLASS |
| Soil Chemical | Soil Organic Carbon | SOC | SoilGrids250m |
|  | Total Nitrogen | TN | SoilGrids250m |
|  | Carbon to Nitrogen Ratio | C:N Ratio | SoilGrids250m |
| Soil Physical | Soil Texture Fractions | Sand, Silt, Clay | SoilGrids250m |
|  | Bulk Density | BD | SoilGrids250m |
|  | Soil Moisture | SM | SoilGrids250m |
|  | Soil pH | pH | SoilGrids250m |
|  | Cation Exchange Capacity | CEC | SoilGrids250m |
| Soil Microbial | Microbial Biomass Carbon | MBC | Gao et al. ^[1]^ |
|  | Microbial Biomass Nitrogen | MBN | Gao et al. ^[1]^ |
|  | Microbial Biomass Phosphorus | MBP | Gao et al. ^[1]^ |
| Topography | Elevation | Derived from DEM | ASTER Global DEM |
|  | Aspect | - | ASTER Global DEM |
|  | Slope | - | ASTER Global DEM |

**Table S8** Comparison of global annual soil respiration estimates by different studies during a defined period.

| **Year published** | **Period** | **Annual soil respiration**  **(Pg C yr^-1^)** | **Spatial resolution** | **Input** | **Approaches** | **Reference** |
| --- | --- | --- | --- | --- | --- | --- |
| 2002 | 1980-2009 | 80.4 | 0.5°×0.5° | Climate data | Regression mode | Raich et al.^[2]^ |
| 2010 | 2008 | 98 | 0.5°×0.5° | Climate data, leaf area index, nitrogen deposition, biome type | Linear model | Bond-Lamberty & Thomson,^[3]^ |
| 2012 | 1980-2009 | 78 | 0.5°×0.5° | Climate data | Process-based model | Hashimoto^[4]^ |
| 2013 | 1970-2008 | 94.4 | 0.5°×0.5° | Climate data, soil organic carbon | Semi-mechanistic, empirically-based model | Chen et al.^[5]^ |
| 2015 | 1965-2012 | 91 | 0.5°×0.5° | Climate data | Process-based model | Hashimoto et al.^[6]^ |
| 2017 | 2001-2009 | 94.8 (2001),  93.8 (2009) | 4 km×4 km | Climate and land-use data | Empirical equations | Adachi et al.^[7]^ |
| 2017 | 1960-2012 | 93.3 | 5'×5' | Climate data, biome type | Artificial neural network model | Zhao et al.^[8]^ |
| 2017 | 2014 | 80.3-108.6 | 0.5°×0.5° | Mean soil temperature, mean soil moisture, total soil carbon, net primary production | GAM models | Hursh et al.^[9]^ |
| 2018 | 1961-2014 | 78.76 | 0.5°×0.5° | Climate, elevation, above-ground biomass, soil organic carbon, and land-use data | Random Forest and exponential models | Jian et al.[10] |
| 2019 | 1961-2011 | 87.9 | 1 km×1 km | Climate data and vegetation index | Quantile regression forest model | Warner et al.^[11]^ |
| 2020 | 2000-2014 | 72.6 | 1 km×1 km | Temperature, moisture and plant productivity data | Biome-specific statistical models | Lu et al.^[12]^ |
| 2021 | 1982–2012 | 85.5 | 0.5°×0.5° | Mean annual temperature, annual precipitation, mean annual relative humidity, leaf area index | Random Forest | Huang et al.^[13]^ |
|  | **1985-2018** | **86.34** | **1 km×1 km** | **Topography, climate, vegetation, soil physics, soil chemistry, soil microbes, and land-use data** | **Nonlinear stepwise regression model** | **This study** |

**Supplementary Table 9** The goodness-of-fit statistics base on Structural equation model in each ecosystem type.

| **Ecosystem types** | **χ/df** | ***P*** | **RMSEA** | **CFI** | **GFI** | **NFI** |
| --- | --- | --- | --- | --- | --- | --- |
| Bareland | 3.355 | 0.067 | 0.057 | 0.998 | 0.998 | 0.997 |
| Grassland | 0.162 | 0.851 | 0.000 | 1.000 | 1.000 | 1.000 |
| Shrubland | 1.399 | 0.237 | 0.025 | 0.999 | 0.999 | 0.998 |
| Needleleaf forest | 1.023 | 0.393 | 0.004 | 1.000 | 0.999 | 0.995 |
| Broadleaf forest | 1.548 | 0.213 | 0.026 | 0.998 | 0.999 | 0.995 |
| Mixed forest | 0.775 | 0.542 | 0.000 | 1.000 | 0.991 | 0.970 |
| Wetland | 0.989 | 0.412 | 0.000 | 1.000 | 0.984 | 0.966 |

**
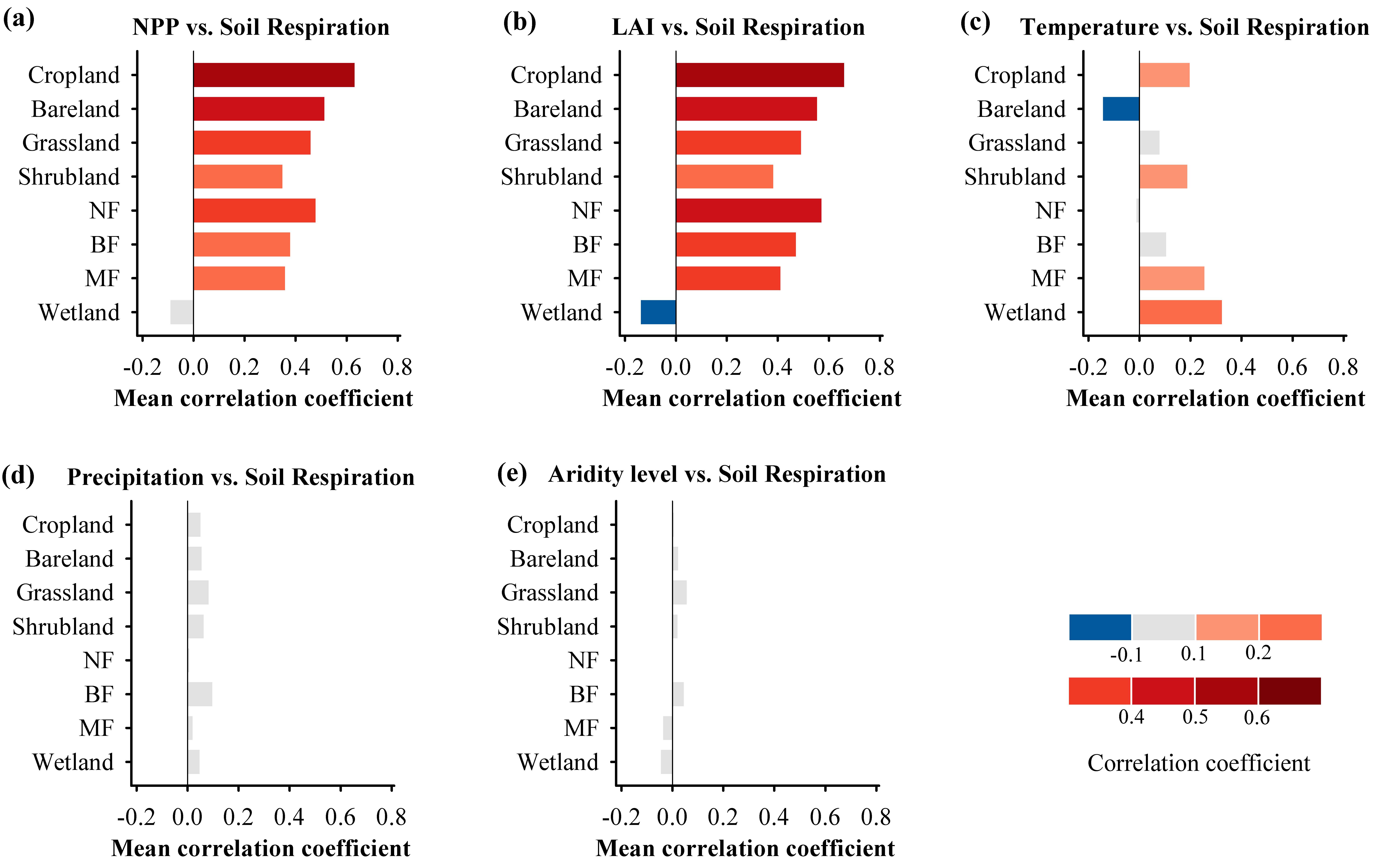
**

**Figure. S1 Relationship of annual soil respiration and its predictors across** **different ecosystem types.** **a-e** The display the mean correlation coefficients of soil respiration with five predictors: NPP, LAI, temperature, precipitation, and aridity level across different ecosystem types. NF: needleleaf forest, BF: broadleaf forest, MF: mixed forest.


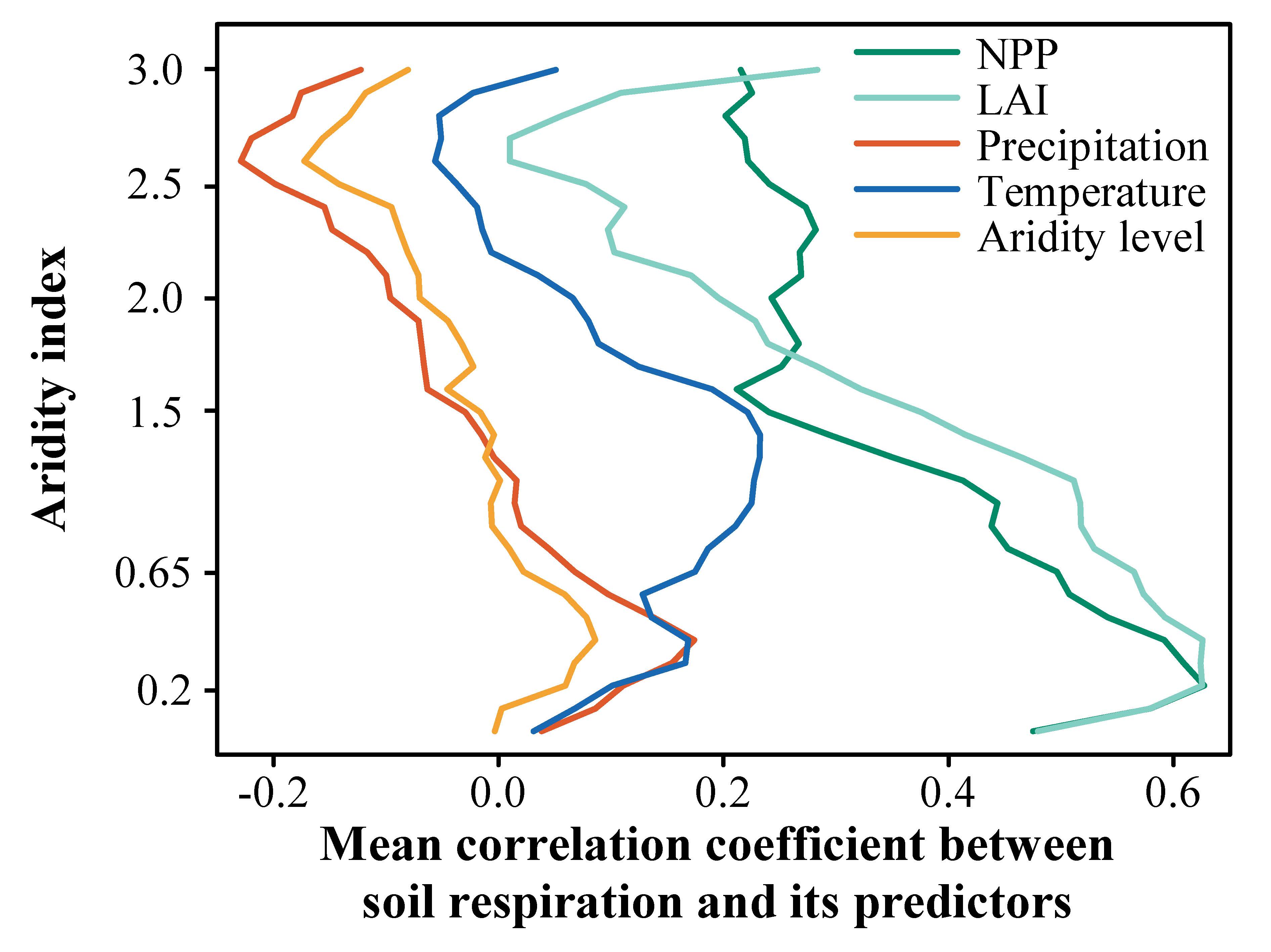


**Figure. S2 Relationship of annual soil respiration and its predictors across an aridity gradient.** Lines represent the mean correlation coefficients of annual soil respiration with aboveground ecosystem productivity (NPP and LAI), precipitation, temperature, and aridity level across an aridity gradient.

**
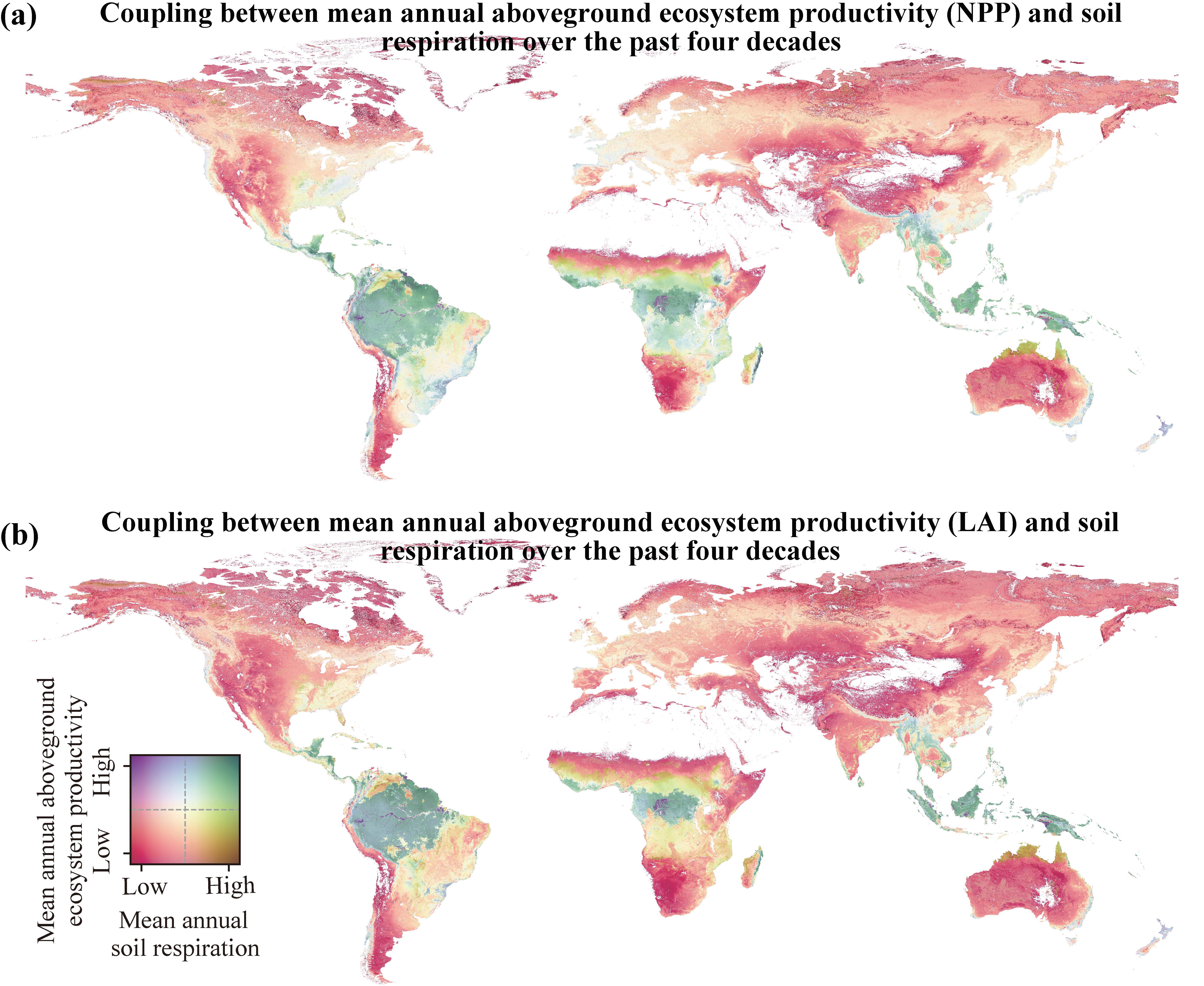
**

**Figure. S3 The spatial coupling between aboveground and belowground ecosystem processes.** **a-b** Global distribution of the overlap between mean annual aboveground productivity (NPP and LAI) and mean annual soil respiration over the past four decades (1985-2018). Low and high values represent the 10th and 90th percentiles of the minimum and maximum values, respectively.


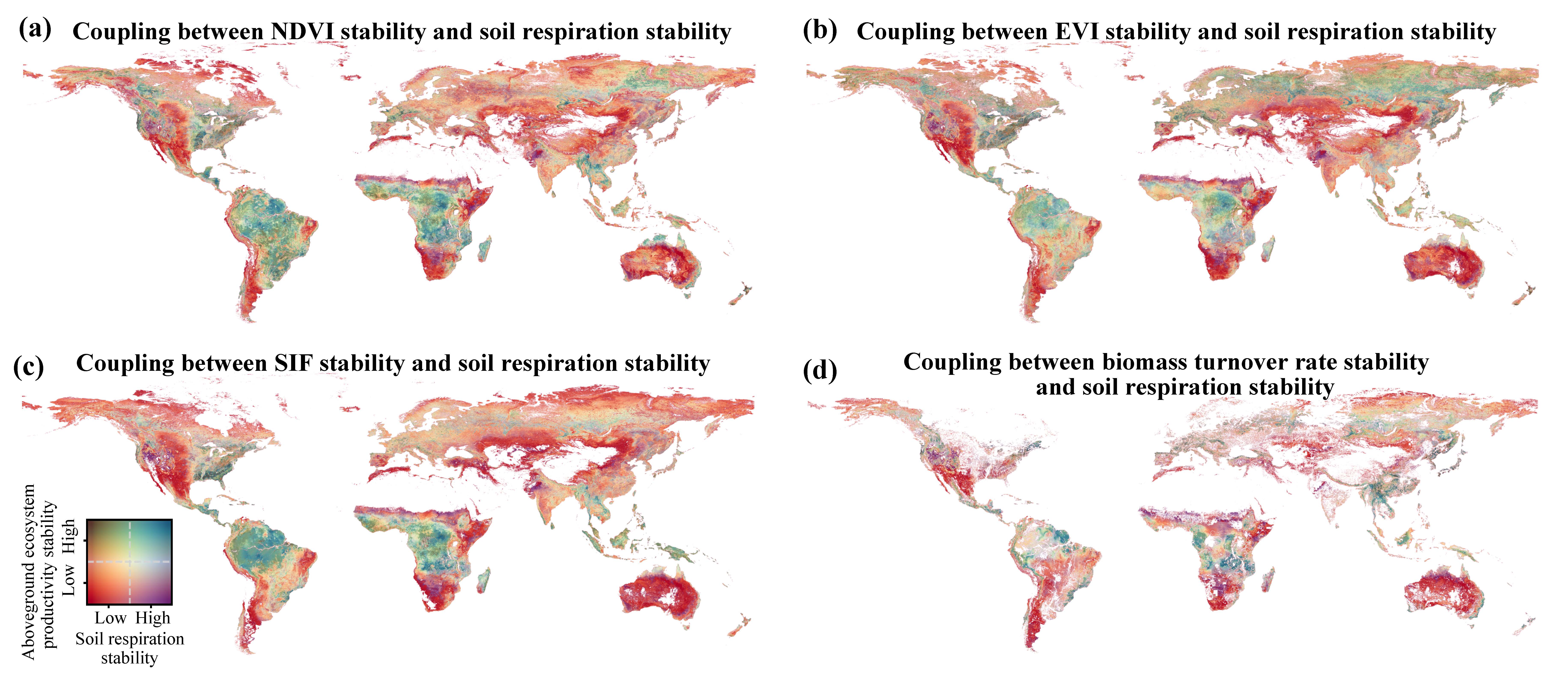


**Figure. S4 Coupling between stability of additional vegetation metrics and soil respiration stability.** **a-d**, Global distribution of the overlap between soil respiration stability and the stability of NDVI (a), EVI (b), solar-induced chlorophyll fluorescence (SIF; c) and biomass turnover rate (d). Low and high values represent the 10th and 90th percentiles of the minimum and maximum values respectively.


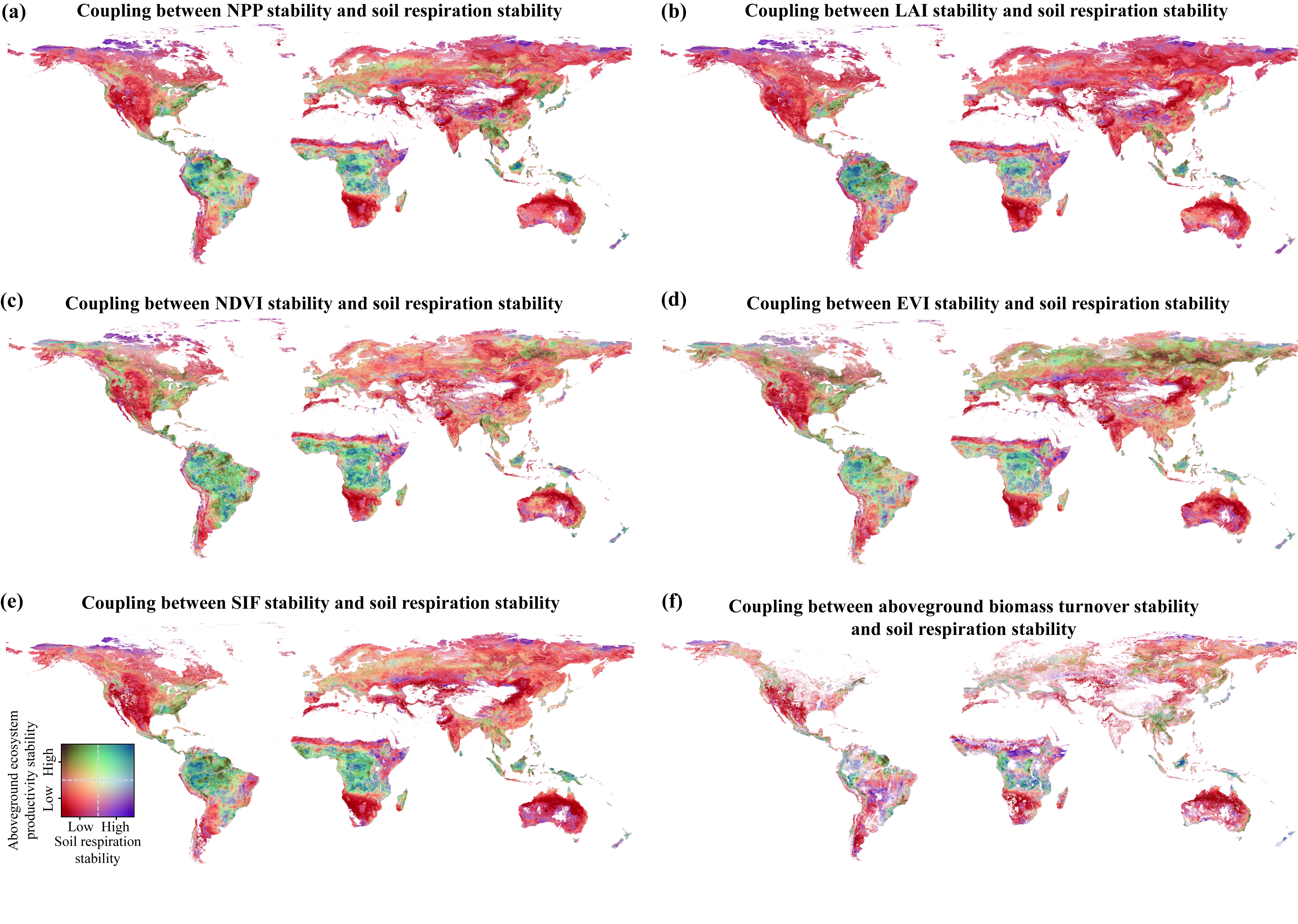


**Figure. S5 Coupling between multiple aboveground productivity stability and soil respiration stability.** Spatial distributions of the coupling between the stability of soil respiration and six aboveground vegetation metrics including (**a**) NPP, (**b**) LAI, (**c**) NDVI, (**d**) EVI, (**e**) SIF, and (**f**) aboveground biomass turnover rate. This soil respiration database are derived from an independent Random Forest reconstruction excluding all vegetation-related predictors to verify the above- and belowground ecosystem coupling. Low and high values represent the 10th and 90th percentiles of the minimum and maximum values, respectively.


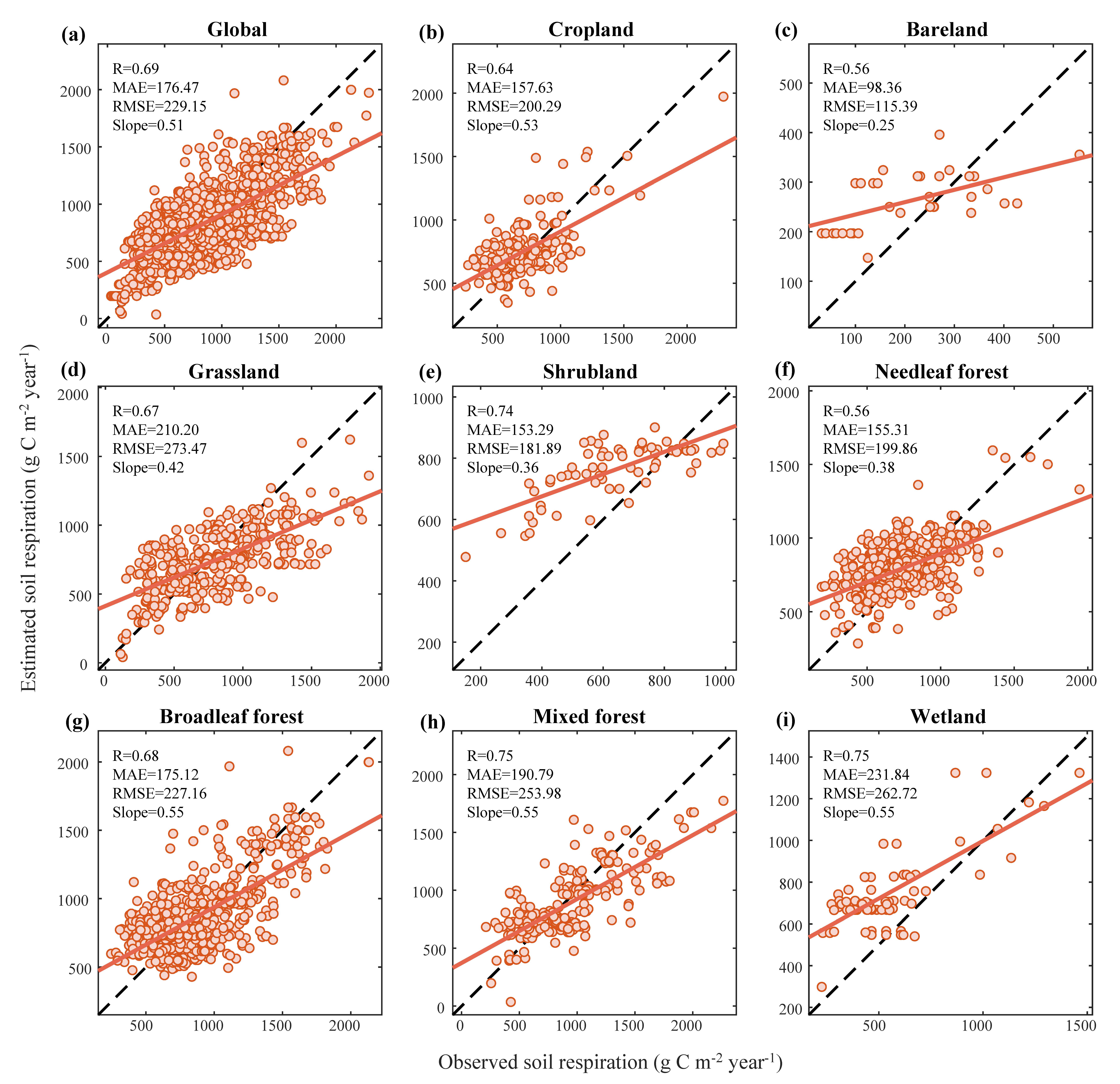


**Figure. S6 Validation of the soil respiration simulation model at global and regional scales**. Scatter plots showing the relationship between observed annual soil respiration and model estimates across the globe and eight major biomes (cropland, bareland, grassland, shrubland, needleleaf forest, broadleaf forest, mixed forest, and wetland). The solid lines represent ordinary least-squares linear regressions, and the dashed lines indicate the 1:1 correspondence. For each panel, the Pearson correlation coefficient (R), Mean Absolute Error (MAE), Root Mean Square Error (RMSE), and regression slope are reported.





**Figure. S7 Comparison of simulated global soil respiration from this and recent studies. a-c** Global distribution of estimated annual mean soil respiration from this study, Lu et al.^[12]^, and Huang et al.^[13]^. **d** Comparison of annual mean soil respiration values from this study and recent studies. **e-f** Pearson correlation test was conducted to investigate the correlation from simulated soil respiration values in this study to Lu et al.^[12]^ and Huang et al.^[13]^. Lines represent the least squares regression fits. Rs: soil respiration.


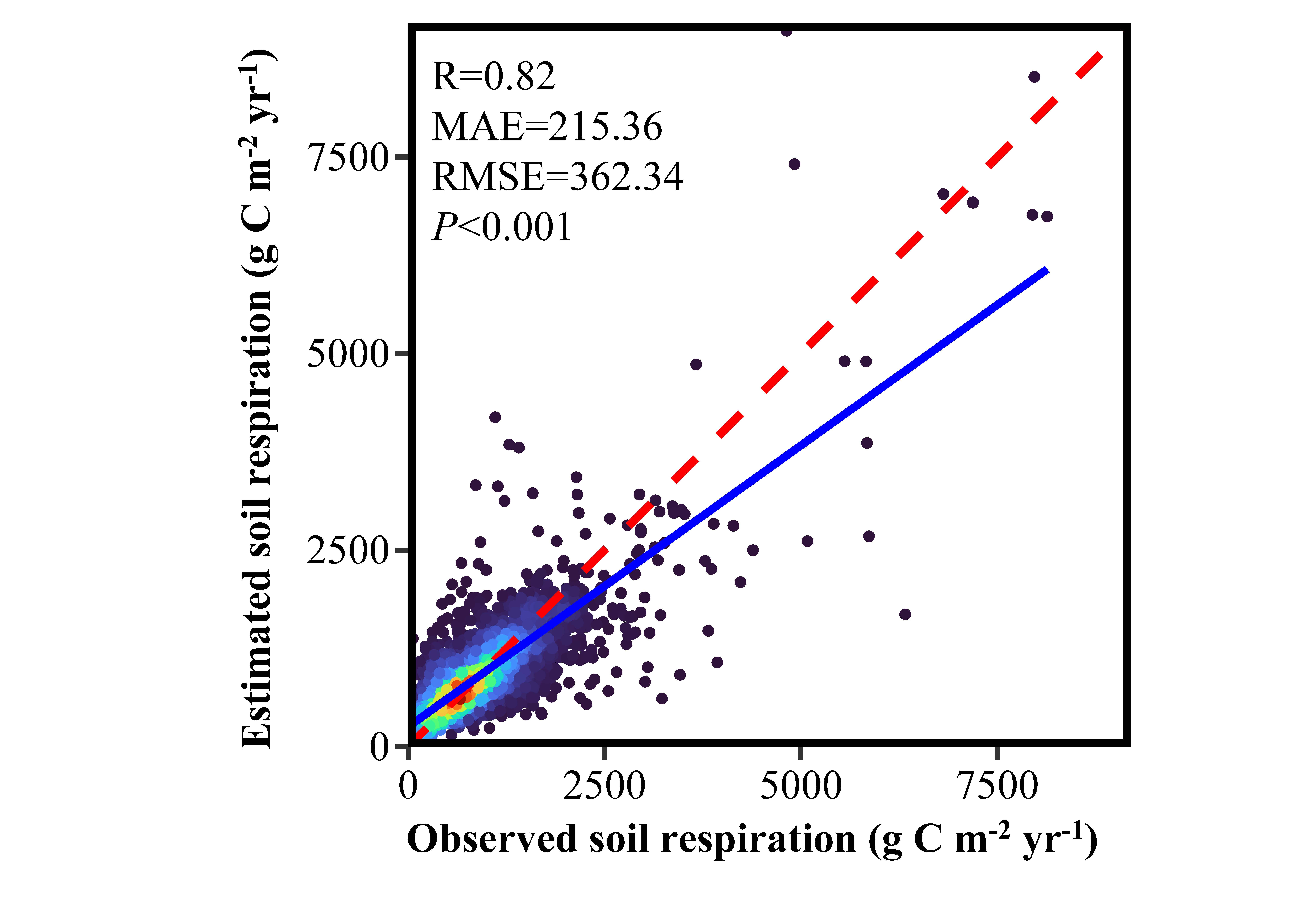


**Figure. S8 Validation of the global soil respiration reconstruction independent of vegetation inputs.** The scatter plot compares observed annual soil respiration from the global dataset with estimated values derived from a Random Forest model that intentionally excluded all vegetation-related predictors. The solid blue line represents the linear regression fit, and the dashed red line indicates the 1:1 correspondence. Statistical metrics, including the Pearson correlation coefficient (R), Mean Absolute Error (MAE), Root Mean Square Error (RMSE), and P value, are provided to evaluate the predictive performance based solely on abiotic environmental variables.


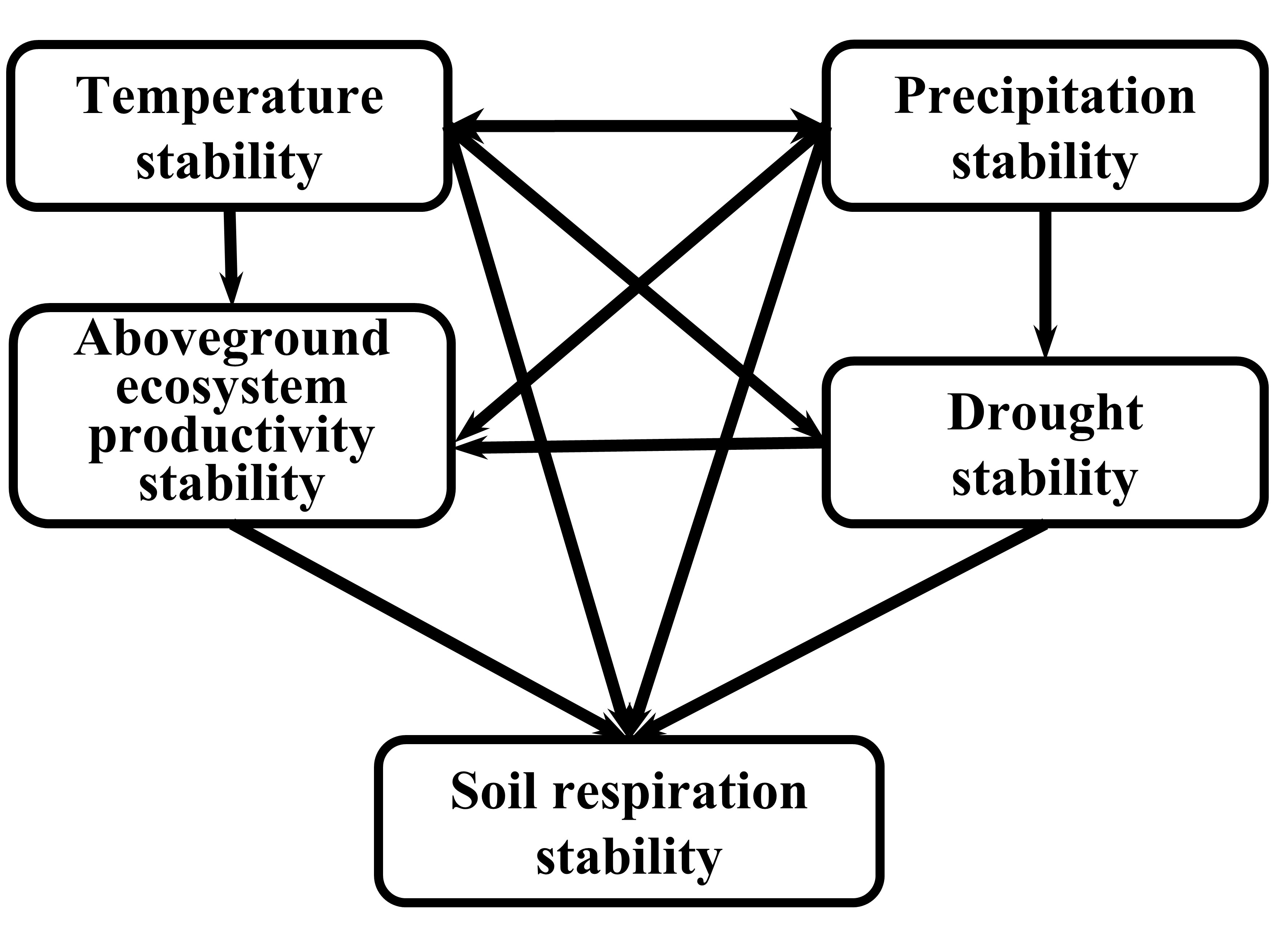


**Figure. S9 A priori Structural equation model used in this study.** A priori Structural equation model illustrating the coupling relationship between aboveground and belowground stability, as well as the role of climate stability (temperature, precipitation, and aridity stability) in this coupling. To prevent overfitting in the model, aboveground ecosystem productivity stability was represented by the first principal component of aboveground ecosystem productivity NPP stability and LAI stability.

**Supplementary references:**

[1] D. C. Gao, E. Bai, S. Y. Wang, et al.,·“Three-dimensional mapping of carbon, nitrogen, and phosphorus in soil microbial biomass and their stoichiometry at the global scale,”·*Global Change Biol*·28·(2022):·6728-6740,·<https://doi.org/10.1111/gcb.16374>.

[2] J. W. Raich, C. S. Potter and D. Bhagawati,·“Interannual variability in global soil respiration, 1980-94,”·*Global Change Biol*·8·(2002):·800-812,·<https://doi.org/10.1046/j.1365-2486.2002.00511.x>.

[3] B. Bond-Lamberty and A. Thomson,·“Temperature-associated increases in the global soil respiration record,”·*Nature*·464·(2010):·579-582,·<https://doi.org/10.1038/nature08930>.

[4] S. Hashimoto,·“A new estimation of global soil greenhouse gas fluxes using a simple data-oriented model,”·*Plos One*·7·(2012):·e41962,·<https://doi.org/10.1371/journal.pone.0041962>.

[5] S. T. Chen, Y. Huang, W. Xie, et al.,·“A new estimate of global soil respiration from 1970 to 2008,”·*Chinese Sci Bull*·58·(2013):·4153-4160,·<https://doi.org/10.1007/s11434-013-5912-1>.

[6] S. Hashimoto, N. Carvalhais, A. Ito, et al.,·“Global spatiotemporal distribution of soil respiration modeled using a global database,”·*Biogeosciences*·12·(2015):·4121-4132,·<https://doi.org/10.5194/bg-12-4121-2015>.

[7] M. Adachi, A. Ito, S. Yonemura, et al.,·“Estimation of global soil respiration by accounting for land-use changes derived from remote sensing data,”·*Journal of Environmental Management*·200·(2017):·97-104,·<https://doi.org/10.1016/j.jenvman.2017.05.076>.

[8] Z. Y. Zhao, C. H. Peng, Q. Yang, et al.,·“Model prediction of biome-specific global soil respiration from 1960 to 2012,”·*Earths Future*·5·(2017):·715-729,·<https://doi.org/10.1002/2016ef000480>.

[9] A. Hursh, A. Ballantyne, L. Cooper, et al.,·“The sensitivity of soil respiration to soil temperature, moisture, and carbon supply at the global scale,”·*Global Change Biol*·23·(2017):·2090-2103,·<https://doi.org/10.1111/gcb.13489>.

[10] J. S. Jian, M. K. Steele, R. Q. Thomas, et al.,·“Constraining estimates of global soil respiration by quantifying sources of variability,”·*Global Change Biol*·24·(2018):·4143-4159,·<https://doi.org/10.1111/gcb.14301>.

[11] D. L. Warner, B. Bond-Lamberty, J. Jian, et al.,·“Spatial predictions and associated uncertainty of annual soil respiration at the global scale,”·*Global Biogeochemical Cycles*·33·(2019):·1733-1745,·<https://doi.org/10.1029/2019gb006264>.

[12] H. B. Lu, S. H. Li, M. N. Ma, et al.,·“Comparing machine learning-derived global estimates of soil respiration and its components with those from terrestrial ecosystem models,”·*Environmental Research Letters*·16·(2021):·054048,·<https://doi.org/10.1088/1748-9326/abf526>.

[13] N. Huang, L. Wang, X. P. Song, et al.,·“Spatial and temporal variations in global soil respiration and their relationships with climate and land cover,”·*Sci Adv*·6·(2020):·eabb8508,·<https://doi.org/10.1126/sciadv.abb8508>.
